# Periostin secreted by activated fibroblasts in idiopathic pulmonary fibrosis promotes tumorigenesis of non-small cell lung cancer

**DOI:** 10.1038/s41598-021-00717-5

**Published:** 2021-10-26

**Authors:** Hiroyuki Yamato, Kenji Kimura, Eriko Fukui, Takashi Kanou, Naoko Ose, Soichiro Funaki, Masato Minami, Yasushi Shintani

**Affiliations:** Department of General Thoracic Surgery, Osaka University Graduate School of Medicine, 2-2-L5, Yamadaoka, Suita, Osaka 565-0871 Japan

**Keywords:** Cancer microenvironment, Lung cancer

## Abstract

Non-small cell lung cancer (NSCLC) patients with idiopathic pulmonary fibrosis (IPF) show poor prognosis. Periostin is an extracellular matrix protein highly expressed in the lung tissues of IPF. This study aimed to investigate the possibility that periostin secreted by fibroblasts derived from IPF lung might affect proliferation of NSCLC cells. Periostin was more highly expressed and secreted by fibroblasts from diseased human lung with IPF (DIPF) than by normal human lung fibroblasts (NHLF). Cocultivation of NSCLC cells with conditioned media (CM) from DIPF increased proliferation of NSCLC cells through pErk signaling, with this proliferation attenuated by periostin-neutralizing antibodies. Knockdown of integrin β3, a subunit of the periostin receptor, in NSCLC cells suppressed proliferation of NSCLC cells promoted by recombinant human periostin and CM of DIPF. On in vivo examination, DIPF promoted tumor progression more than NHLF, and knockdown of integrin β3 in NSCLC cells suppressed tumor progression promoted by DIPF. Fibroblasts derived from surgical specimens from IPF patients also increased secretion of periostin compared to those from non-IPF patients. Periostin secreted from IPF-activated fibroblasts plays critical roles in the proliferation of NSCLC cells. The present study provides a solid basis for considering periostin-targeted therapy for NSCLC patients with IPF.

## Introduction

Idiopathic pulmonary fibrosis (IPF) is a chronically progressive, intractable disease characterized by fibroblast activity in the lung parenchyma and showing a poor prognosis^1^. IPF is a risk factor for the development of non-small cell lung cancer (NSCLC)^2^. The development of lung cancer (LC) is one of the main causes of death in IPF patients, along with respiratory failure and acute exacerbation (AE) due to the progression of the IPF itself^3^. NSCLC with IPF (LC-IPF) is more aggressive than NSCLC without IPF (LC-non-IPF)^4^. Indeed, patients with LC-IPF display even worse prognosis than patients with LC-non-IPF^5^. Whereas some reports have stated that the pathology of IPF relates to increasing malignancy of NSCLC in IPF patients, details of the underlying mechanisms remain unclear in many aspects. In addition, few treatment options are available for patients with LC-IPF in clinical practice. Major NSCLC therapies such as cytotoxic chemotherapy, molecularly targeted therapies, immune checkpoint inhibitors and radiotherapy are difficult to use for patients with LC-IPF because of the high risk of AE of IPF^6^. New treatments are therefore needed for NSCLC patients with IPF. Furthermore, the development of new targeted therapies requires elucidation of the relationships between IPF and NSCLC.

A significant number of IPF patients display risk factors also associated with NSCLC, so multiple common genetic, molecular, and cellular processes appear to connect lung fibrosis with NSCLC^5,7^. Several growth factors such as transforming growth factor β (TGF-β)^8^ and platelet-derived growth factor^9^ are chronically overexpressed in fibrosis and LC. Significant overexpression of the secreted mucin protein has been found in LC-IPF^10^. IPF-activated fibroblasts release molecules such as TGF-β and cytokines to advance the pathology of IPF, and many of these cytokines are also involved in the development of NSCLC^7,11^. The Wnt/β-catenin pathway and the TGF-β pathway activated in IPF-activated fibroblasts each play a role as signals promoting NSCLC progression and tissue infiltration^12,13^. Therapeutic agents targeting pathways that are activated in common for these two diseases have recently been attracting attention. We have reported that pirfenidone, an anti-fibrotic agent for IPF, inhibits not only fibroblast activity, but also the crosstalk between cancer cells and fibroblasts^14,15^. Nintedanib, an inhibitor of tyrosine kinase receptors, exerts antitumor effects as well as antifibrotic effects on IPF^16^. Identifying a brand-new common molecule for these two pathologies and clarifying the function of the molecule on cancer could thus be key to overcoming LC-IPF.

Periostin is an extracellular matrix protein that binds to cells via integrin molecules to promote cell proliferation^17^. Periostin is deeply involved in the abnormal activity of interstitial fibrosis, which is the main pathology of IPF^18^. Inhibition of periostin expression suppresses bleomycin-induced lung fibrosis in vivo^19^. Further, high expression of periostin is observed in many carcinomas, including NSCLC, and is a factor associated with poor prognosis^20,21^. Overexpression of periostin promotes proliferation of breast cancer cells both in vitro and in vivo^22^. Periostin thus offers a potential therapeutic target for LC-IPF. However, whether periostin expression in activated fibroblasts of IPF is involved in the tumor progression of LC-IPF remains unclear.

In this study, we identified that periostin was highly expressed in IPF-activated fibroblasts apart from tumor lesions in LC-IPF. Here, we reveal the effects of periostin secreted by fibroblasts derived from IPF lung on NSCLC cell proliferation, and propose the possibility of a new therapeutic strategy targeting periostin for NSCLC patients with IPF.

## Results

### Patients with LC-IPF show worse prognosis than those with LC-non-IPF

**Figure 1:**
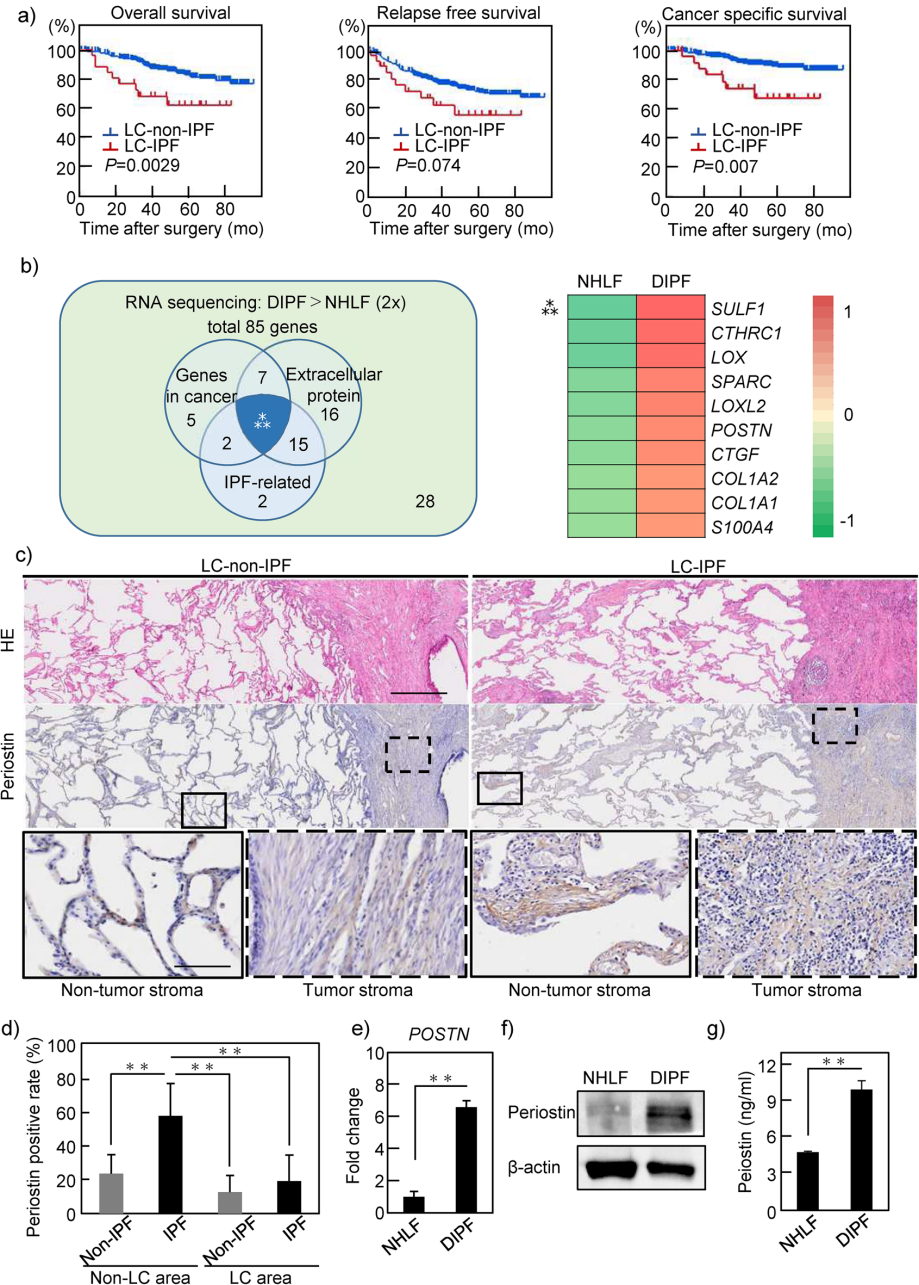
Clinical significance of idiopathic pulmonary fibrosis (IPF) in non-small cell lung cancer (NSCLC) patients. (**a**) Patients who underwent NSCLC resection in our hospital were divided into LC-IPF (n = 512) and LC-non-IPF groups (n = 26). Clinical outcome data including overall survival, relapse-free survival and cancer-specific survival among groups were compared using Kaplan–Meier methods. (**b**) Based on RNA-seq results, a total of 85 genes were upregulated with a more than twofold change in DIPF compared to NHLF (one fibroblast cell line from each group examined, analysis performed once). The Venn diagram in the left panel shows genes related to cancer selected from ‘The Atlas of Genetics and Cytogenetics in Oncology and Haematology, genes related to extracellular proteins, and genes related to IPF. The numbers in the Venn diagram represent the number of genes corresponding to the criteria. Ten genes are identified as common genes within these categories. Details of these 10 identified genes are shown in the right panel. Ratios of NHLF to DIPF (left, the darker the green, the smaller the value) and DIPF to NHLF (right, the darker the red, the larger the value) are shown. (**c**) HE staining and periostin immunostaining of pathological specimens from patients with resected LC of LC-IPF and LC-non-IPF, respectively. Solid and dashed squares show enlarged images of non-tumor and tumor stroma areas, respectively. Scale bars, 0.5 mm in upper and middle panels; 100 μm in lower panels. (**d**) LC-non-IPF (n = 26) and LC-IPF (n = 26) specimens quantified for the area of periostin-positive tissue relative to the area of tissue. (**e**–**g**) Expression of periostin in NHLF and DIPF cells as compared by RT-PCR (**e**), WB (**f**), and ELISA (**g**). Statistical significance was tested with the log-rank test (**a**) or the Mann–Whitney U test (**d**,**e**,**g**). **P < 0.01. LC-IPF, NSCLC patient with IPF; LC-non-IPF, NSCLC patient without IPF; mo, months; RNA-Seq, ribonucleic acid sequencing; WB, Western blot; NHLF, normal human lung fibroblast; DIPF, diseased human lung fibroblast-IPF; *SULF1*, sulfatase 1; *CTHRC1*, collagen triple helix repeat containing 1; *LOX*, lysyl oxidase; *SPARC*, secreted protein acidic and cysteine rich; *LOXL2*, lysyl oxidase-like 2; *POSTN*, periostin; *CTGF*, connective tissue growth factor, *COL1A2*, collagen type I alpha 2 chain; *COL1A1*, collagen type I alpha 1 chain; *S100A4*, S100 calcium-binding protein A4.

To clarify the clinicopathological characteristics of LC-IPF patients, we compared the clinical and pathological factors of LC-IPF and LC-non-IPF patients (Supplementary Table 1). Of the 538 patients included in this study, 26 were pathologically diagnosed with LC-IPF (4.8%). Consistent with the disease background of IPF^23,24^, LC-IPF patients were more likely to be older men and heavy smokers. LC-IPF patients had larger tumor diameters and higher level of cytokeratin 19 fragment (Cyfra) than LC-non-IPF patients. In addition, LC-IPF had higher maximum standardized uptake value (SUVmax) in positron emission tomography-computed tomography (PET-CT). This value is clinically used to evaluate the metabolic activity and malignancy of tumors. Furthermore, western blot (WB) for pErk expression was performed using frozen specimens obtained from patients, which showed a high level of pErk expression in LC-IPF as compared to LC-non-IPF samples (n = 3 each), suggesting that cell proliferation signaling is more highly activated in tumors with IPF (Supplementary Fig. 1a, Supplementary Table 2). Overall survival was significantly decreased in LC-IPF patients as compared to LC-non-IPF patients (Fig. 1a). In addition, both relapse-free survival and cancer-specific survival (CSS) were also decreased in LC-IPF patients as compared to LC-non-IPF patients. Notably, CSS showed a significant difference between these two groups (Fig. 1a).

### Periostin was more highly expressed in DIPF than in NHLF

We hypothesized that factors involved in fibroblast activation in IPF might also be involved in the malignant transformation of NSCLC cells, and screened for factors common to these two diseases. RNA-sequencing analyses were performed in normal human lung fibroblasts (NHLF) and diseased human lung fibroblasts derived from idiopathic pulmonary fibrosis (DIPF) (one fibroblast cell line for each group). RNA sequence results revealed 761 genes that were more highly expressed in DIPF than in NHLF. From a total of 761 genes, 85 (Supplementary Fig. 2a) were selected based on the following conditions: RNA expression in DIPF was more than twice that in NHLF and the number of fragments per kilobase of exon per million reads mapped (FPKM) of DIPF was greater than 100. Pathway analysis of the 85 genes was performed using the Metascape program (https://metascape.org) (Supplementary Fig. 2b). Those results showed that pathways related to the extracellular matrix (ECM) were at the top. From 85 genes, we selected cancer-related genes according to a public database. In those genes, we focused on secretory proteins, because secretory proteins are one of the main factors secreted by non-cancerous fibroblasts to act on nearby cancer cells. As a result, we extracted 10 candidate genes (Fig. 1b). Among these genes, we focused on *periostin*, which is already considered a poor prognostic factor in other carcinomas.

To identify the localization of periostin expression in LC-IPF patients, 26 cases of LC-IPF were analyzed by immunostaining (Fig. 1c). In LC-IPF, periostin staining was stronger in both cancerous and non-cancerous areas than in LC-non-IPF. Notably, the most strongly staining areas were the stroma of fibrotic lesions of non-cancerous areas in LC-IPF patients. The positive periostin staining rate was 12.7% in the cancerous area and 23.5% in the non-cancerous area in LC-non-IPF patients, and 19.0% in the cancerous area and 57.2% in the non-cancerous area in LC-IPF patients (Fig. 1d). In both groups of patients, periostin was more highly expressed in the non-cancerous area than in the cancerous area. Notably, the highest positive rate was found in the interstitial area of IPF in LC-IPF. RT-PCR and WB analyses of frozen clinical samples showed that periostin expression was greatest in the non-LC area (IPF area) of LC-IPF (Supplementary Fig. 3, Supplementary Tables 2, 3). Similar to the results of immunostaining for clinical samples, in vitro examination showed higher expression of *periostin* mRNA by DIPF than by NHLF (Fig. 1e). Immunoblots showed higher expression of periostin by DIPF than by NHLF, and enzyme-linked immunosorbent assay (ELISA) showed higher periostin concentrations in conditioned media (CM) from DIPF than in that from NHLF (Fig. 1f,g). Expression levels of *collagen type I alpha 1 chain* (*COL1a1*) and *TGF-β2* mRNA were significantly higher from DIPF than from NHLF, but no significant difference was observed in *actin alpha 2* (*ACTA2*) (Supplementary Fig. 4).

Taken together, these results indicated that periostin, which was highly secreted by IPF-derived fibroblasts, could be involved in the malignant transformation of LC-IPF.

### Periostin contained in the CM of DIPF promotes LC cell line proliferation

**Figure 2:**
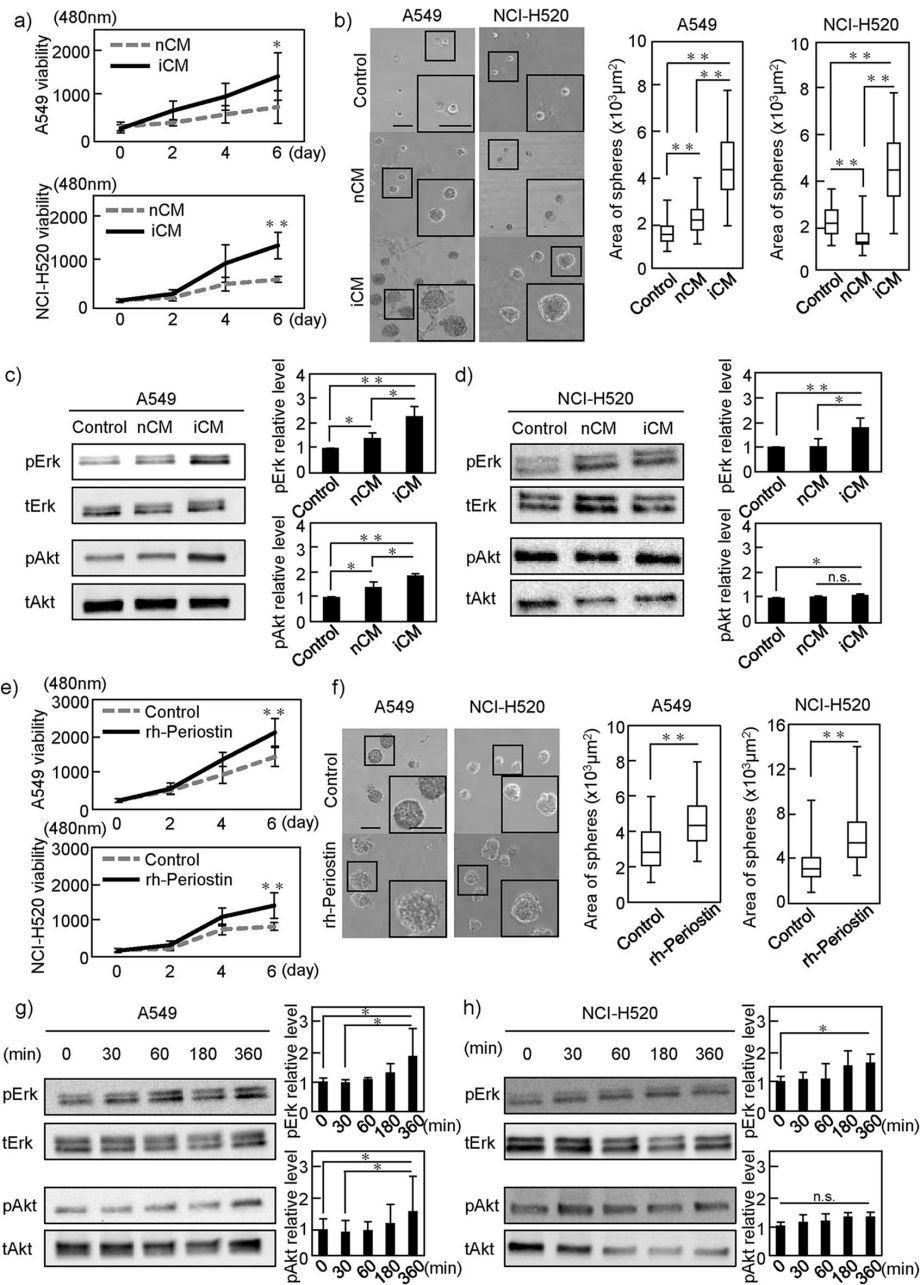
Effects of conditioned media (CM) of fibroblasts and recombinant human periostin on NSCLC cell lines. (**a**) A549 (NCI-H520) cells were cultured in CM of NHLF (nCM) and DIPF (iCM) cells, respectively. Cell growth in 2D was evaluated. (**b**) A549 (NCI-H520) cells were cultured for 6 days in 3D Matrigel containing nCM or iCM, and the area of the sphere was measured. (**c**,**d**) A549 (NCI-H520) cells were stimulated with CMs for 6 h. Lysates of cells were probed with anti-pErk and pAkt. Left images show representative images of WB. Right figures show quantification of signal intensity. (**e**) A549 (NCI-H520) cells were cultured in DMEM with or without rh-periostin (200 ng/ml) and cell growth in 2D was evaluated. (**f**) A549 (NCI-H520) cells were cultured for 6 days in 3D Matrigel containing DMEM with or without rh-periostin (200 ng/ml). The area of the sphere was measured. (**g**,**h**) A549 (NCI-H520) cells were stimulated with rh-periostin for the indicated time, then signals of pErk and pAkt were analyzed by WB. Statistical significance was tested with the Mann–Whitney U test. Solid squares show enlarged images and scale bars, 100 μm in (**b**) and (**f**). *P < 0.05, **P < 0.01. n.s., non-significant; nCM, normal human lung fibroblast-derived conditioned media; iCM, diseased human lung fibroblast-IPF-derived conditioned media; rh-periostin, recombinant human periostin; Erk, extracellular signal-regulated Kinase; WB, western blot.

LC-IPF patients had a large tumor as a clinicopathological characteristic (Supplementary Table 1). We therefore examined the effects of CM containing various secretions from NHLF (nCM) and DIPF (iCM) on the growth of NSCLC cells. Since most histological types of LC-IPF are NSCLC, such as adenocarcinoma and squamous cell carcinoma, we conducted experiments using two cell lines, the A549 lung adenocarcinoma cell line and the NCI-H520 squamous cell carcinoma cell line. Using two-dimensional (2D) culture conditions, iCM increased cancer cell growth (Fig. 2a). When both A549 and NCI-H520 cells were cultured under three-dimensional (3D) culture conditions using Matrigel, iCM also increased the area of the tumor sphere compared to nCM (Fig. 2b). Furthermore, in these two cancer cell lines, the growth signal of pErk, which is reportedly associated with periostin-induced proliferation^25^, was more strongly activated in iCM than in nCM (Fig. 2c,d). The PI3K/Akt pathway was activated by iCM stimulation in A549 cells, but was almost unchanged in NCI-H520 cells.

To confirm whether the proliferation of A549 or NCI-H520 cells is increased by periostin, we used recombinant human (rh-) periostin. When cancer cells were cultured in serum-free media with or without rh-periostin, rh-periostin increased the growth of cancer cells under both 2D and 3D conditions (Fig. 2e,f). In addition, rh-periostin upregulated pErk signaling in both A549 and NCI-H520 cells in a time-dependent manner (Fig. 2g,h).

### Periostin secreted by DIPF promotes tumorigenesis of NSCLC cells in vivo

**Figure 3:**
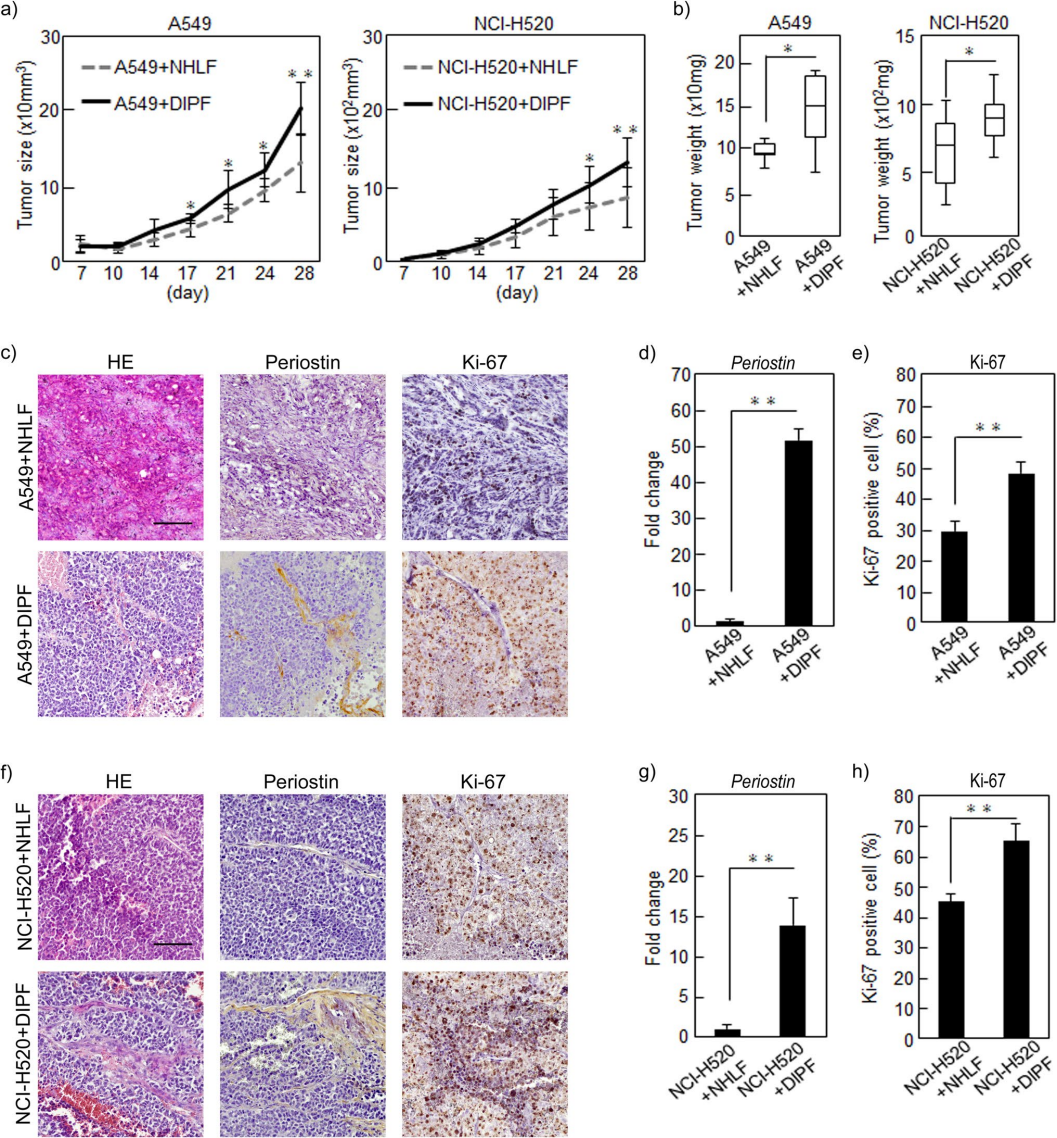
Effects of fibroblasts on NSCLC cells in vivo. A549 or NCI-H520 cells were mixed with NHLF or DIPF in a 5:1 ratio, then co-injected subcutaneously into nude mice (A549: n = 5; NCI-H520: n = 6) that were sacrificed 28 days after co-injection. (**a**) Tumor size after co-injection of cells was plotted over time. (**b**) Tumor weight 28 days after co-injection. (**c**,**f**) Xenograft tumors of (**c**) A549 and (**f**) NCI-H520 cells stained with HE, anti-periostin or anti-Ki-67 antibody. (**d**,**g**) Expression of periostin in xenograft tumors (A549, NCI-H520 cells) with NHLF and in xenograft tumors with DIPF compared by RT-PCR. (**e**,**h**) Ki-67-positive cells were counted in xenograft tumors (A549, NCI-H520 cells). Results are shown as mean ± SD expressed as the percentage of positively stained cells compared with the total number of cells. Statistical significance was tested with the Mann–Whitney U test. Solid squares show enlarged images and scale bars, 100 μm in (**c**,**h**). *P < 0.05, **P < 0.01. NHLF, normal human lung fibroblast; DIPF, disease human lung fibroblast-IPF; HE, hematoxylin and eosin.

We evaluated the effects of DIPF on NSCLC cell proliferation in vivo using a subcutaneous tumor model in immunodeficient mice. We mixed NSCLC cells (A549 or NCI-H520) with NHLF or DIPF and injected these mixtures into the flanks of nude mice (n = 5). Tumor sizes of cancer cells with DIPF were significantly increased from day 17 on A549 cells and from day 24 on NCI-H520 compared to cancer cells with NHLF (Fig. 3a). In both A549 and NCI-H520 cells, tumors derived from cancer cells with DIPF were heavier than those with NHLF (Fig. 3b). Results of RT-PCR analysis of xenograft tumors showed that periostin expression was also higher in tumor cells with DIPF than in those with NHLF in both LC cell lines (Fig. 3d,g). Furthermore, immunostaining analysis of A549 xenograft tumors showed that periostin was highly expressed in DIPF around tumor cells and the Ki-67-positive rate of tumor cells with DIPF was significantly higher than that of tumor cells with NHLF (Fig. 3c,e). These results suggest that DIPF may increase the proliferative potential of NSCLC cells. The results of this immunostaining analysis were similar in NCI-H520 (Fig. 3f,h). These results suggested that periostin secreted by DIPF promoted tumorigenesis of cancer cells in vivo.

### Periostin neutralizing antibody attenuated proliferation of NSCLC cells promoted by DIPF

**Figure 4:**
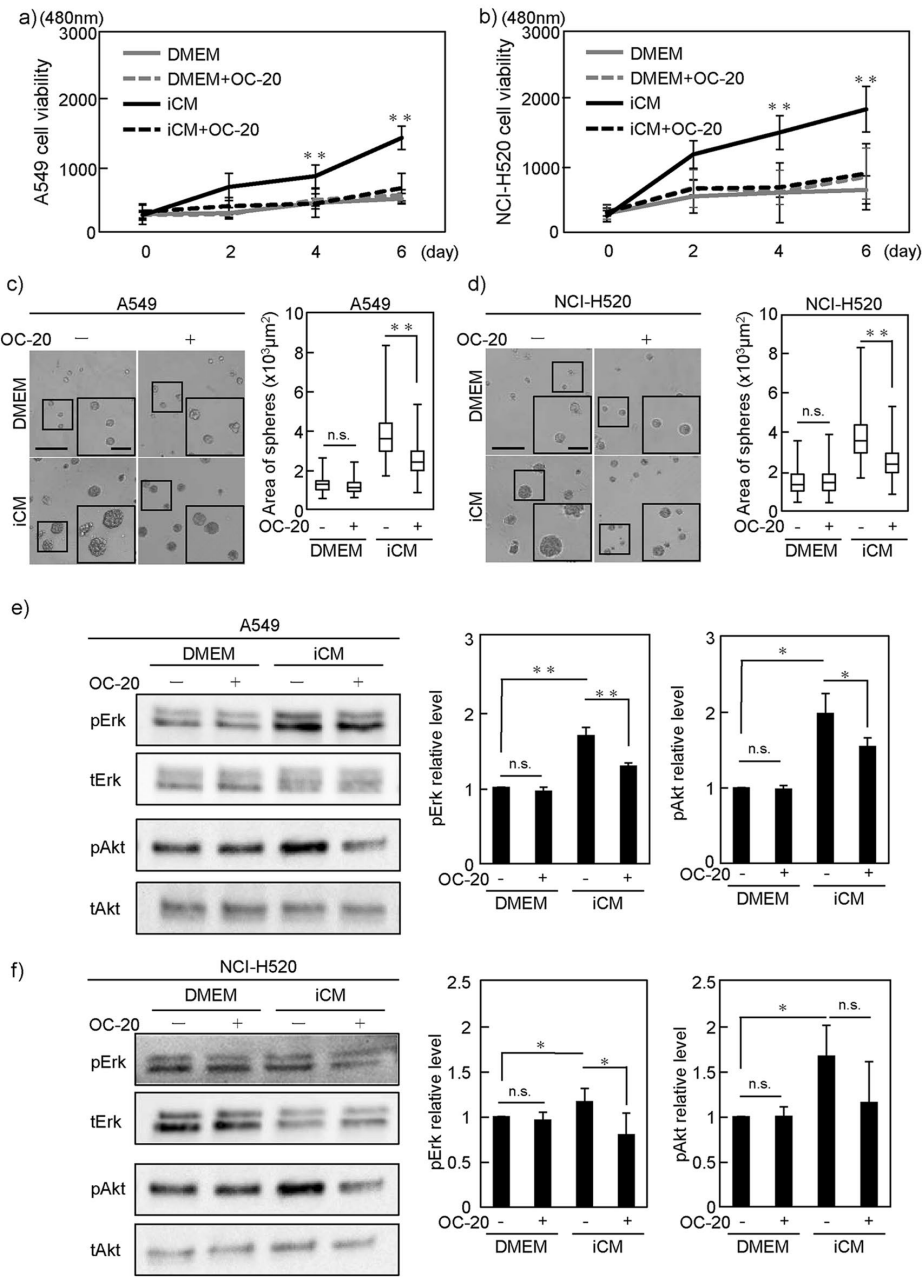
Effect of periostin-neutralizing antibody on proliferation of NSCLC cells. (**a**,**b**) A549 (NCI-H520) cells cultured in iCM with or without periostin-neutralizing antibody, OC-20 (2 µg/ml). Cell growth in 2D was evaluated. (**c**,**d**) A549 (NCI-H520) cells were cultured for 6 days with or without OC-20 (2 μg/ml) in 3D Matrigel containing iCM. Volumes of the spheres were then measured. (**e**,**f**) A549 (NCI-H520) cells were cultured iCM with or without OC-20 (2 µg/ml) for 6 h. Phosphorylation of Erk and Akt was evaluated by WB. DMEM was used as control in all experiments. Statistical significance was tested using the Mann–Whitney U test. Solid squares show enlarged images and scale bars, 100 μm in (**c**,**d**). *P < 0.05, **P < 0.01. n.s., non-significant; iCM, diseased human lung fibroblast IPF-derived conditioned media; Erk, extracellular signal-regulated kinase; WB, Western blot.

To examine the effect on cancer cells of a loss of the periostin secreted by fibroblasts, we examined whether adding OC-20, a neutralizing antibody of periostin, to iCM would cancel the effects of periostin on NSCLC cells. Whereas OC-20 did not affect cell proliferation of A549 or NCI-H520 cells in serum-free culture media under 2D culture conditions, OC-20 significantly attenuated iCM-induced upregulation of NSCLC cell proliferation (Fig. 4a,b). Under 3D culture conditions, OC-20 inhibited the increase in cell proliferation induced by iCM for both NSCLC cell types (Fig. 4c,d). Similar to this result, OC-20 reduced the upregulated pErk signaling in A549 or NCI-H520 cells when cultured in iCM (Fig. 4e,f). These results suggest that OC-20 neutralized periostin in iCM, resulting in the inhibition of cell proliferation induced by iCM.

### Knockdown of ITB3 attenuated periostin-induced cell proliferation in NSCLC cells

**Figure 5:**
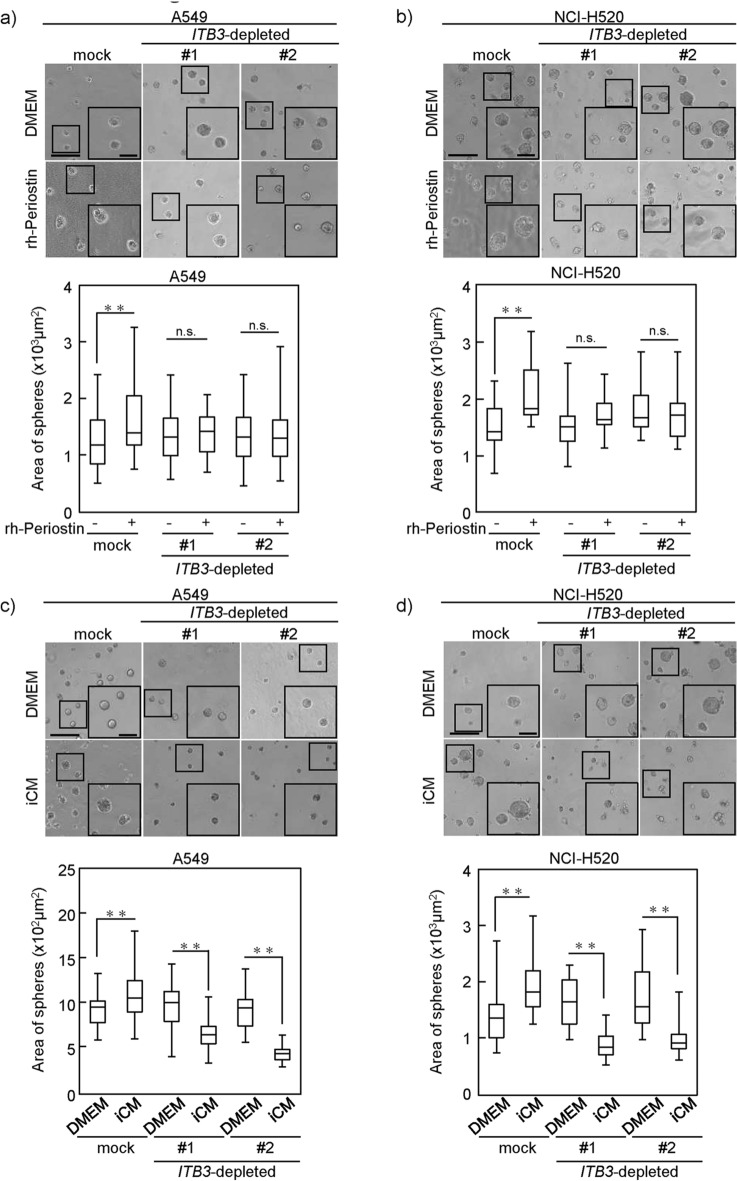
Effect of periostin receptor silencing on NSCLC cells. (**a**,**b**) ITB3 knockdown A549 (**a**) or NCI-H520 (shITB3#1 and shITB#2) (**b**) cells and Mock-A549 or Mock-NCI-H520 cells were cultured for 6 days in 3D Matrigel containing DMEM with or without rh-periostin (200 ng/ml) and the area of the sphere was measured and evaluated. Upper panels show representative images of cell spheres and lower panels show results for sphere size. (**c**,**d**) The shITB3-A549 and shITB3-NCI-H520 (#1 and #2) cells were cultured for 6 days in 3D Matrigel containing DMEM or iCM with or without OC-20 (2 µg/ml), and the area of the sphere was measured. Mock-A549 or Mock-NCI-H520 cells were used as control. Statistical significance was tested with the Mann–Whitney U test. Solid squares show enlarged images and scale bars, 100 μm in (**a**–**d**). *P < 0.05, **P < 0.01. n.s., non-significant; ITB3, integrin β3; rh-periostin, recombinant periostin; iCM, diseased human lung fibroblast-IPF-derived conditioned media.

Integrin β3 (ITB3) is a subunit of the periostin receptor^26^. We generated A549 or NCI-H520 cells transfected with *ITB3* small hairpin (sh) RNA, constitutively reducing *ITB3* expression (Supplementary Fig. 5a,b). To more specifically confirm the effect of periostin on NSCLC cells, we examined the effects of stimulating these *ITB3*-depleted A549 or NCI-H520 cells with rh-periostin and iCM on cell proliferation in 3D culture. Cell proliferation of mock A549 cells, which normally express *ITB3*, was clearly increased by rh-periostin and iCM (Fig. 5a,b). On the other hand, cell proliferation of *ITB3*-depleted A549 cells was not increased despite stimulation by rh-periostin or iCM. Interestingly, cell proliferation of *ITB3*-depleted NSCLC cells was reduced in iCM as compared to serum free culture media (Fig. 5c,d).

These results suggested that periostin stimulation played a major role in enhancing proliferation in iCM, and that iCM was a less suitable environment for cell proliferation than serum free culture media alone when periostin stimulation was eliminated.

### Knockdown of *ITB3* attenuated tumorigenesis of NSCLC cells promoted by DIPF in vivo

**Figure 6:**
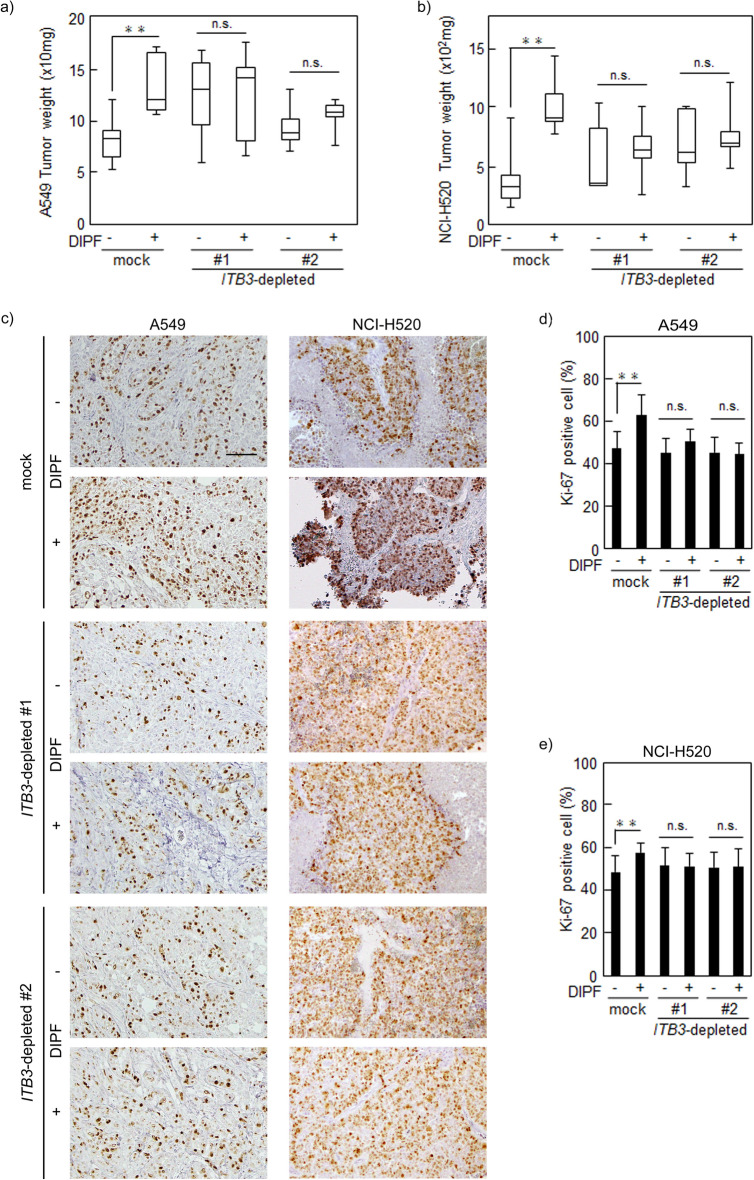
Effect of periostin receptor silencing on NSCLC cells in vivo. *ITB3*-depleted A549 or NCI-H520 cells were mixed with or without DIPF in a 5:1 ratio, co-injected subcutaneously into nude mice (n = 5), and sacrificed 28 days after co-injection. (**a**,**b**) Mice with *ITB3*-depleted A549 (NCI-H520) cells with or without DIPF co-injection were sacrificed 28 days after co-injection and tumor weight was measured. (**c**) Sections were prepared from xenograft tumors of A549 (NCI-H520) stained with anti-Ki-67 antibody. (**d**,**e**) Ki-67-positive cells were counted in xenograft tumors of A549 (**d**) and NCI-H520 (**e**). Results are shown as mean ± SD expressed as the percentage of positively stained cells compared with the total number of cells. Solid squares show enlarged images and scale bars, 100 μm in (**c**). *P < 0.05, **P < 0.01. n.s., non-significant; ITB3, integrin β3; DIPF, diseased human lung fibroblasts with IPF.

To evaluate whether *ITB3*-depleted NSCLC cells were affected by DIPF in tumorigenesis in vivo, we injected mock or *ITB3*-depleted NSCLC cells with DIPF in a 5:1 ratio into the right flank of nude mice and NSCLC cells into the left flank (n = 5). No significant difference in tumor size or weight was seen between mock A549 and *ITB3*-depleted A549 cells. Mock A549 cells with DIPF were significantly larger and heavier from day 21 in tumor size and tumor weight as compared to those without DIPF (Supplementary Fig. 6a, Fig. 6a). On the other hand, in *ITB3*-depleted A549 cells, no difference in tumor size or weight was seen with and without DIPF up to day 28 (Supplementary Fig. 6a, Fig. 6a). Similar results were observed for NCI-H520 cells (Supplementary Fig. 6b, Fig. 6b). Immunohistochemically, periostin was highly expressed in cancer stroma derived from mice injected with NSCLC cells combined with DIPF. Mock A549 or NCI-H520 cells with DIPF had more Ki67-positive cells than those without DIPF, but *ITB3*-depletion suppressed increased Ki67-positive NSCLC cells when injected with DIPF (Fig. 6c–e).

These results suggested that periostin secreted by DIPF played an important role in NSCLC cell tumorigenesis in vivo through the periostin receptor, integrin β3.

### Fibroblasts from LC-IPF lung had higher expression and greater secretion of periostin

Finally, we evaluated whether expression and secretion of periostin in fibroblasts derived from LC-IPF lung were higher than those from LC-non-IPF lung. According to the methods described, fibroblasts were isolated from surgical specimens of LC-non IP patients (NLfs, n = 5) and LC-IPF patients (LC-IPFfs, n = 6). Fibroblasts from an IPF patient without LC (non-LC-IPFfs, n = 1) were used as a reference for comparison with the former two kinds of fibroblasts. The clinicopathological characteristics of the patients from whom fibroblasts were isolated are shown in Supplementary Table 4. IPFfs showed higher levels of *periostin* expression than NLfs (Supplementary Fig. 7a). The mRNA expression levels of *COL1a1* and *TGF-β2* were significantly higher for IPFfs than for NLfs, but no significant difference was observed for *ACTA2*, consistent with the analyses using NHLF and DIPF (Supplementary Fig. 7b–d). Furthermore, ELISA showed that CM from IPFfs contained periostin at higher concentrations than that from NLfs (Supplementary Fig. 7e). *Periostin* expression in pairs of IPFfs and fibroblasts obtained from tumor tissues (IPF-CAFs) from the IPF patients (n = 6, Supplementary Table 4) was examined by RT-PCR. IPFfs tended to have a higher level of periostin expression, though the difference was not significant (Supplementary Fig. 7f).

## Discussion

We showed that patients with LC-IPF had a poor prognosis and LC-IPF had higher SUVmax in FDG-PET, indicating that LC-IPF had higher malignant potential than LC-non-IPF. Although the risk of NSCLC is eight times higher in IPF patients than in the general population and IPF is associated with poor prognosis in IPF-LC patients^27^, the detailed molecular mechanisms remain unclear. IPF comprises a heterogeneous group of disorders characterized by lung damage as a result of lung fibroblast proliferation, interstitial inflammation, and fibrosis^28^. In this study, we identified periostin as a secreted protein involved in both IPF and NSCLC. Periostin is a matricellular protein secreted by fibroblasts that interacts with various cell-surface integrin molecules. This protein has been reported to increase cell survival, angiogenesis, invasion, metastasis, and chemoresistance in various cancers^29–31^, so we focused on periostin derived from IPF-activated fibroblasts.

We showed that cocultivation of NSCLC cells with CM of DIPF increased proliferation of NSCLC cells, and these effects were attenuated by periostin-neutralizing antibodies as well as by knockdown of *ITB3*, a subunit of the periostin receptor, indicating that periostin secreted from DIPF promoted tumorigenesis of NSCLC cells. In vivo examinations found that DIPF promoted tumor progression more than NHLF, and knockdown of *ITB3* in NSCLC cells suppressed tumor progression promoted by DIPF. Our data showed that periostin affected NSCLC cell growth through pErk and pAkt signaling. This result is consistent with previous reports of functional analysis of periostin in a mouse NSCLC model^31^. Increased proliferative capacity is a major phenotype that characterizes malignant transformation of cancer. These data suggested that periostin secreted from activated fibroblasts in the IPF lung might correlate with carcinogenesis and progression of NSCLC.

Periostin was highly expressed in clinical samples from NSCLC patients with IPF as compared to those without IPF, especially in non-cancerous areas. We performed primary culture of fibroblasts from surgical specimens of patients and showed that fibroblasts from IPF lung also displayed greater secretion of periostin than those from non-IPF lung. Periostin is reportedly required for maximal proliferation of normal lung fibroblasts, and IPF lung fibroblasts retain this activity^32^. Periostin seems to play a critical role in the pathogenesis of pulmonary fibrosis, and thus offers a promising therapeutic target for IPF. Nanri et al. found that cross-talk between the TGF-β and periostin axes is necessary for pulmonary fibrosis, and created an integrin αvβ3 receptor antagonist that attenuated fibroblast activation and has potential as a therapeutic agent for IPF^33^. Periostin is reportedly elevated during AE of IPF^34^, suggesting that periostin may contribute to such AE. These data suggest that periostin represents a potential target in IPF progression and AE as well as in NSCLC.

We analyzed the function of periostin in NSCLC cells by stimulating NSCLC cells with CM of activated fibroblasts in vitro and co-injecting NSCLC cells and activated fibroblast into nude mice in vivo. These results indicated the effect of increased proliferation of NSCLC cells by periostin secreted from activated fibroblasts. Many researchers have demonstrated that cancer-associated fibroblasts (CAFs) secrete periostin, which promotes cancer initiation and progression^35^. Although bone marrow-derived mesenchymal stem cells, hematopoietic stem cells, epithelial cells (epithelial-mesenchymal transition), and endothelial cells (endothelial-mesenchymal transition) are all considered possible predecessors of CAFs, a common theory of the origins of CAFs points to resident tissue fibroblasts^36^. Activated fibroblasts in IPF lung may thus represent one origin of CAFs in IPF-LC. On the other hand, periostin is reportedly found in serum^37^ and even in exosomes in vitro^38^. We also considered that periostin secreted by activated fibroblasts is most likely to be contained in exosomes and reach cancer cells hematogenously, but further experiments should be performed to elucidate the exact mechanisms of the interaction.

Periostin-targeted therapy for cancer has recently been developed. The periostin epitope recognized by monoclonal antibody OC-20 is a binding site for the integrins αvβ3 and αvβ5, localized in the second fasciclin-1 domain of the protein. An in vivo study found that this antibody significantly inhibited tumor growth and angiogenesis^39^. Furthermore, eight splicing variants of human periostin have been reported and characterized^40^, and the application of blocking antibodies specifically targeting cancer-specific variants of periostin may be valid for cancer treatment^41^. An engineered peptide antagonist against periostin also binds to periostin at an integrin-binding site and inhibits periostin function, and thus is expected to see use in peptide therapies to reduce periostin-induced cancer progression and chemoresistance^42^. Whereas patients with LC-IPF have few treatment options because of the risk of IPF progression following cancer treatment, a periostin-targeted therapeutic agent for LC-IPF may be clinically beneficial for both IPF and NSCLC in the future.

This study has several limitations. First, it was not possible to statistically compare periostin expression between fibroblasts from IPF lungs derived from clinical samples of patients with LC and those from patients without LC because of ethical issues. On the other hand, periostin expression in cultured fibroblasts from IPF lung sections of a patient without LC was similar to that seen in those obtained from lungs of LC patients, thus we concluded that there is no significant difference between patients with and without lung cancer. Second, we were not able to clarify how periostin secreted by fibroblasts is actually transported from activated fibroblasts in non-cancerous areas to NSCLC cells in the human tumor microenvironment. Nevertheless, it is considered that periostin secreted by non-tumor stroma in IPF-LC plays an important role in tumor progression, especially in LC development, because LC is known to often arise de novo from an IPF lung.

In conclusion, this is the first study to demonstrate that periostin derived from activated fibroblasts in IPF lung plays a critical role in the proliferation of NSCLC and that inhibition of periostin-receptor interactions attenuated the aggressiveness of NSCLC with IPF. Our findings provide novel insights into the roles of periostin in LC-IPF and indicate that periostin offers a potentially useful target for the treatment of both IPF and NSCLC.

## Methods

### Patients

We analyzed clinical and pathological data from 538 patients who underwent lung resection to treat NSCLC at Osaka University Hospital between 2011 and 2016. Pathological diagnosis of the resected specimen determined the presence or absence of IPF. Tumors were staged according to the 7th edition of the Union for International Cancer Control TNM staging system. Resected specimens for histological examination were fixed in 10% formalin and routinely processed for paraffin embedding. Tissues were sectioned into slices with a thickness of 4 µm. The protocol for this study was approved by the ethics review board at Osaka University Hospital (approval no. 18518) in accordance with the Declaration of Helsinki, and written informed consent was waived because of the retrospective design. We also applied Opt-out method to obtain consent on this study which was approved by the ethics review board.

### Cell lines

NHLF and DIPF were purchased from Lonza (Walkersville, MD, USA) and maintained using a Fibroblast Growth Media kit (Lonza, Basel, Switzerland) in a humidified incubator with 5% CO_2_ at 37 °C. A549 and NCI-H520 cells were purchased from the American Type Culture Collection (ATCC, Manassas, VA, USA) and grown in Dulbecco's modified Eagle’s medium (DMEM) (Sigma-Aldrich, St. Louis, MO, USA) with 10% fetal bovine serum (FBS) (Sigma-Aldrich) and 100 U/mL penicillin/streptomycin (Wako Pure Chemical Industries, Osaka, Japan) under the same conditions as above.

### RNA sequencing of fibroblasts

Total RNA was extracted from fibroblasts (NHLF and DIPF) using an RNeasy^®^ Mini Kit (#74106; Qiagen, Valencia, CA, USA). Preparation of a next-generation sequencing library was performed using a SMARTer^®^ Stranded Total RNA Sample Prep Kit–Pico Input Mammalian (TaKaRa, Shiga, Japan). Sequencing was performed on an Illumina HiSeq 2500 platform in 75-base single-end mode with Illumina Casava 1.8.2 software for base calling. Sequenced reads were mapped to the human reference genome sequence (hg19) using TopHat v.2.0.13 software, in combination with Bowtie 2 v.2.2.3 and SAMtools v.0.1.19. Fragments per kilobase of exon per million mapped fragments were calculated using Cufflinks v.2.2.1. Raw data files for RNA-sequencing have been deposited in the NCBI Gene Expression Omnibus (GEO) database under the accession code GSE185259. Of the 26,257 genes analyzed, 85 genes were upregulated with a more than twofold change in DIPF compared to NHLF. The Venn diagram shows genes related to cancer selected from ‘The Atlas of Genetics and Cytogenetics in Oncology and Haematology (http://atlasgeneticsoncology.org/index.html)’, genes related to extracellular protein, and genes related to IPF. Numbers in the Venn diagram represent the number of genes corresponding to that criterion.

### Immunohistochemistry

Immunohistochemistry was performed as previously described^43^. All sections stained with periostin were assessed as the percentage of the area positive for periostin compared to the total tissue area using ImageJ software. The labeling index (labeling frequency as a percentage) of Ki67 staining was calculated using the following formula: (number of positive nuclei/total number of cells) × 100. All Results were quantified by counting more than 100 tumor cells in five randomly selected areas per specimen.

### Antibodies and reagents

Antibodies used for Western blotting and immunofluorescence were as follows: anti-periostin (ab14041; Abcam, Cambridge, UK); anti-β-actin (PM053; BML, Nagoya, Japan); anti-integrin β-3 (66952-1-lg; Proteintech, Manchester, UK); anti-phospho-Erk1/2 (#4370; Cell Signaling, Beverly, MA, USA); anti-Erk1/2 (#4695; Cell Signaling); anti-phospho-Akt (#9271; Cell Signaling); anti-Akt (#9272; Cell Signaling); and anti-Ki67 (M7240; Dako, Carpinteria, CA, USA). Reagents used in a cell proliferation assay were as follows: OC-20 (#AG-20B-6000PF-C100; AdipoGen Life Sciences, San Diego, CA, USA), and recombinant human (rh-) periostin (548-F2; R&D Systems, Minneapolis, MN, USA).

### RNA extraction and quantitative real-time (RT)-PCR

Total RNA was extracted from A549, NCI-H520, NHLF and DIPF cells using the RNeasy Mini Kit (#74106; Qiagen, Valencia, CA, USA), and complementary DNAs were synthesized from 1.0 μg of total RNA with the PrimeScrip TM RT mastermix (RR036A; Takara Bio, Shiga, Japan). RT-PCR was performed using Luna Universal qPCR Master Mix (M3003L; New England BioLabs, Ipswich, UK). *Glyceraldehyde 3-phosphate dehydrogenase* (*GAPDH*) was used as an endogenous reference gene. The following primer sequences were used: for human *periostin*, F 5′-GTCTTTGAGACGCTGGAAGG-3′ and R 5′- AGATCCGTGAAGGTGGTTTG-3′; for human *integrin β3* (*ITB3*), F 5′-AGATGCGAAAGCTCACCAGT-3′ and R 5′-TCCGTGACACACTCTGCTTC-3′, for human *GAPDH*, F 5′-ACCCAGAAGACTGTGGATGG-3′ and R 5′-TTCTAGACGGCAGGTCAGGT-3′. All experiments were performed three times independently. Data shown represent the averages of results from the three experiments.

### Western blot analysis

Monolayers of cultured cells were treated under the indicated conditions, and proteins were extracted with RIPA buffer (#9806; Cell Signaling). Cell extracts were resolved and immunoblotted as described previously^44^. Quantification of immunoblots was performed using ImageJ. All experiments were performed three times independently.

### Enzyme-linked immunosorbent assay (ELISA) for periostin

Cell lines and primary cultures of NHLF and DIPF, respectively, were seeded in 6-well plates (4 × 10^5^ cells/well). After 12 h of incubation, medium (10% FBS) was removed, and cells were fed with serum-free DMEM (2 ml). Conditioned media were collected after 72 h, and periostin was quantified using ELISA according to the protocol from the manufacturer (Human Periostin ELISA kit; Abcam). Absorbance was measured at 450 nm in a microplate spectrophotometer, and periostin levels were quantified with a calibration curve using a rh-periostin standard (ELISA kit). All experiments were performed three times independently.

### Production of conditioned media of fibroblasts

CM were obtained from NHLF (nCM) and DIPF (iCM). Fibroblasts resulting from both cultures were replated at 3 × 10^6^ cells per 10-cm dish and incubated in DMEM (10% FBS) for 12 h. Attached cells were washed three times with phosphate-buffered saline, and the medium was replaced with serum-free DMEM. The supernatant was collected after centrifugation at 1000×*g* and stored at − 80 °C. Each CM was melted just before use in the experiment.

### Cell proliferation assays

A549 and NCI-H520 cells were seeded in 96-well plates at 5 × 10^2^ and 1 × 10^3^ cells/well, respectively. Cells were starved for 24 h, then used in cell proliferation assays. Periostin-neutralizing antibody (OC-20) was added into the culture medium at the same time cells were starved. The culture medium was changed on days 2 and 4. On days 0, 2, 4, and 6, according to the CyQUANT^®^ NF protocol, culture medium was discarded and 100 μl of 1 × dye-binding solution was dispensed into each well. The fluorescence intensity of each sample was measured 30 min later using a fluorescence microplate reader (SH-9000Lab; Hitachi High-Tech Science Corporation, Tokyo, Japan). All experiments were performed three times independently.

### Three-dimensional (3D) cultures of A549 and NCI-H520 cells

The 3D Matrigel (Corning, Fisher Scientific, UK) cultures of A549 and NCI-H520 cells and were obtained as previously described^45^. Briefly, 40 μl of Matrigel was mounted on a round coverslip and incubated for 15 min at 37 °C to solidify the gel. A549 cells (5 × 10^3^ cells) and NCI-H520 cells (1 × 10^4^ cells) suspended in 1 ml of growth medium containing 2% Matrigel were incubated in DMEM (FBS10%) for 24 h. Cultured cells in the Matrigel were divided to 3 groups in which media were replaced with DMEM, DMEM + rh-periostin (200 ng/ml), or serum-free CM, and changed every 2 days. Seven days after medium was exchanged for DMEM + rh-periostin or CM, cells were fixed, photographed, and the area of the sphere was evaluated with ImageJ. All experiments were performed two times independently.

### Knockdown of ITB3 expression by shRNA

A vesicular stomatitis virus G protein-pseudotyped lentivirus encoding shRNA targeting human integrin β3 (ITB3, SHCLNV-NM_000212; Sigma-Aldrich) and a nontargeting shRNA were purchased from Sigma-Aldrich. A549 and NCI-H520 cells were seeded in 12-well plates (3 × 10^4^ cells/well). The CM was DMEM (FBS 10%). At 24 h after seeding, DMEM (10% FBS) was replaced with DMEM (FBS-) adjusted to 5 times the amount of virus of the seeded cells and polybrene (10 µg/ml), and centrifuged at 1200×*g* for 30 min. Four hours later, cells were replaced with DMEM (10% FBS). Cells were passaged at 90% confluency. From 24 h after passage, cells were selected in DMEM (FBS 10%) with puromycin (1 µg/ml). Finally, stable cells with decreased expression of ITB3 were established.

### Animal studies

A549 or NCI-H520 cells were mixed with NHLF or DIPF (5:1) co-injected subcutaneously into 4- to 5-week-old female BALB/cAJcl-nu/nu mice (CLEA Japan, Tokyo, Japan) (5 mice per group). Tumors were measured 2 times per week and tumor volume was calculated using the following equation: tumor volume = D/2 × d^2^, where D was the long diameter and d was the short diameter. Mice were sacrificed 28 days after cells injection, then xenograft tumors were removed for weight measurement and pathological analysis. To examine the effect of periostin secreted from DIPF in vivo, 4- to 5-week-old female BALB/cAJcl-nu/nu mice were implanted subcutaneously with A549 and NCI-H520 cells (Mock; shITB3#1, #2) mixed with DIPF or without (n = 5 mice per group). After injection, the same method described above was used. All animal studies were approved by the Osaka University Animal Experiment Committee (approval no. 28-007-009) and performed in accordance with the Osaka University Regulations on Animal Experiments. All animal work was performed and reported according to the ARRIVE guidelines.

### Frozen clinical samples

Frozen clinical specimens were obtained in an aseptic manner from patients undergoing a pulmonary resection procedure and stored in RNAlater™ Stabilization Solution (AM7024, Thermo Fisher Scientific, MA, USA) at 4 °C for 24 h followed by liquid nitrogen, then used for RT-PCR or WB analysis. Frozen clinical specimens from LC-non-IPF (n = 4), LC-IPF (n = 4), and cancer-free IPF patients (n = 1) were utilized in the present study, with both cancerous and non-cancerous portions used as specimens from patients with LC. After collection, the cancerous and non-cancerous tissues were clearly separated and stored separately. For RT-PCR, all samples were usable. However, for WB, one sample each from the LC-non-IPF and LC-IPF specimens could not be used because of lack of quantity. Thus, WB was investigated using tissues obtained from three LC-non-IPF and three LC-IPF (n = 3) patients, as well as one patient without cancer. WB analysis of pErk was performed only using the cancerous part from the same cases. The clinicopathological characteristics of the patients are shown in Supplementary Table 2 (WB) and Supplementary Table 3 (RT-PCR). The protocol for this study was approved by the Institutional Review Board for Clinical Research at Osaka University Hospital (approval no. 10026, 18528), while written informed consent for surgical intervention was obtained from each patient.

### Primary culture of human fibroblasts

Primary human fibroblasts were isolated from specimens of lung tissue surgically resected from NSCLC patients. Normal lung-derived fibroblasts (NLfs) were harvested from non-cancerous areas of LC-non-IPF patients (n = 5), and IPF-derived fibroblasts (IPLfs) were harvested from non-cancerous areas of LC-IPF patients (n = 6) and from a patient without NSCLC (n = 1), then isolated and cultured. Briefly, normal lung and lung with IPF obviously distant from cancerous tissue was obtained aseptically from patients with NSCLC undergoing pulmonary resection. The institutional review board at Osaka University Hospital approved the study protocol (approval no. 10257), and written informed consent for surgical intervention was obtained from each patient prior to resection. The method of isolating fibroblasts from resected lung and culturing them in primary culture has been described previously^44^. All experiments using primary cultures of fibroblasts were performed between passages 2 and 7.

### Statistical design and data analysis

The log-rank test and Mann–Whitney U test were used to compare results, as appropriate. Values of P < 0.05 were considered significant.

## Supplementary Information

Supplementary Information 1.

Supplementary Information 2.
