# Supplementary material for: Periostin secreted by activated fibroblasts in idiopathic pulmonary fibrosis promotes tumorigenesis of non-small cell lung cancer
Source: Sci Rep. 2021 Oct 26;11:21114. doi: 10.1038/s41598-021-00717-5 (PMC8548404; doi:10.1038/s41598-021-00717-5)
Supplement: Supplementary file 1 — Supplementary Information 1. [file 41598_2021_717_MOESM1_ESM.docx]

**Periostin secreted by activated fibroblasts in idiopathic pulmonary fibrosis promotes tumorigenesis of non-small cell lung cancer**

Hiroyuki Yamato, Kenji Kimura, Eriko Fukui, Takashi Kanou, Naoko Ose, Soichiro Funaki, Masato Minami, and Yasushi Shintani

**Supplementary information**

**Supplemental Figure 1. pErk expression in clinical samples.**

(a) pErk expression was assessed in frozen human samples (LC area of non-IPF or IPF) using WB (n=3 per group).

WB, western blot; IPF, idiopathic pulmonary fibrosis; LC, lung cancer

**
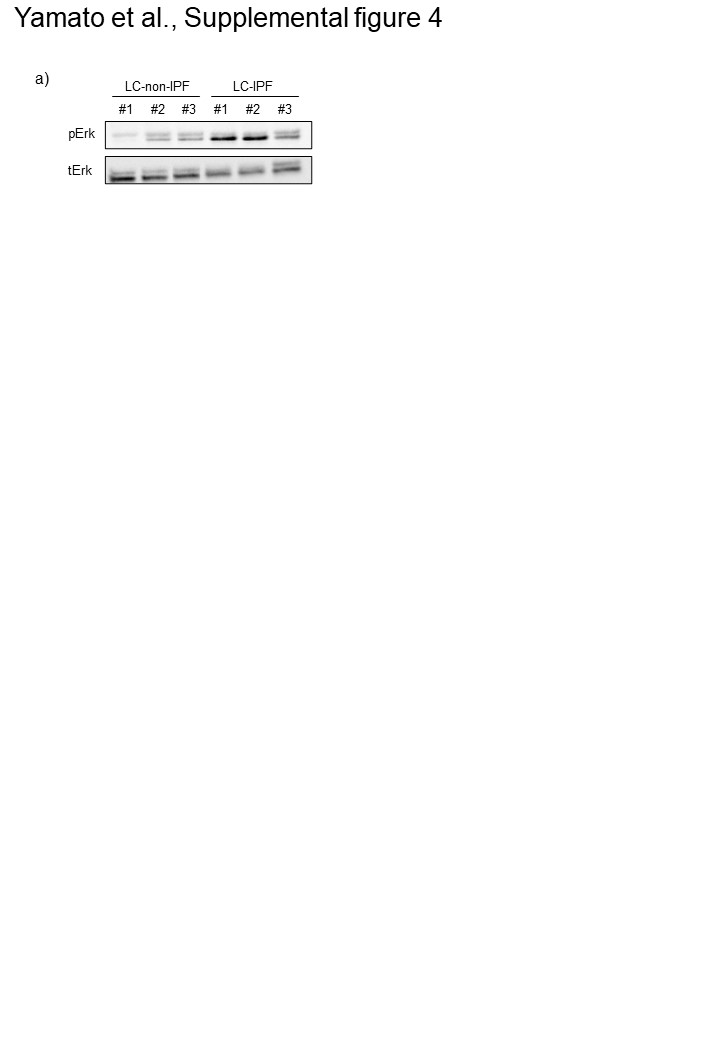
**

**Supplemental Figure 2. RNA sequencing for evaluation of NHLF and DIPF differences.**

(a) Based on RNA sequencing results, 85 genes that showed greater than two-fold expression of DIPF as compared to NHLF, and with a value for fragments per kilobase of exon per million reads mapped (FPKM) in DIPF greater than 100. The 85 genes are listed in detail. (b) Pathway analysis of the 85 genes shown in (a) was performed using the Metascape program (https://metascape.org). Periostin was included in the top two pathways.

NHLF, normal human lung fibroblasts; DIPF, diseased human lung fibroblasts-IPF


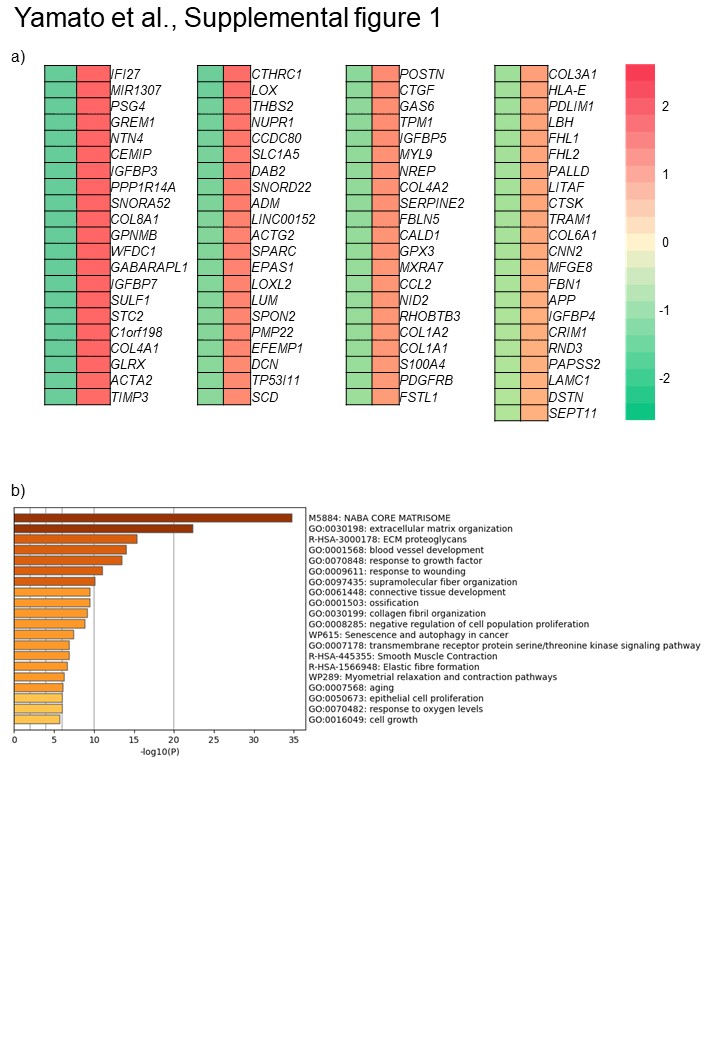


**Supplemental Figure 3. Periostin expression in clinical samples.**

(a, b) Frozen human samples were assessed for periostin expression (non-LC area of non-IPF or IPF, LC area of non-IPF or IPF, and non-LC-IPF) using (a) RT-PCR (n=4; per group, non-LC-IPF; n=1) and (b) WB (n=3; per group, non-LC-IPF; n=1).

Statistical significance was examined with a Mann-Whitney U test. n.s., non-significant.

RT-PCR, real time-PCR; WB, western blotting; IPF, idiopathic pulmonary fibrosis; LC, lung cancer

**
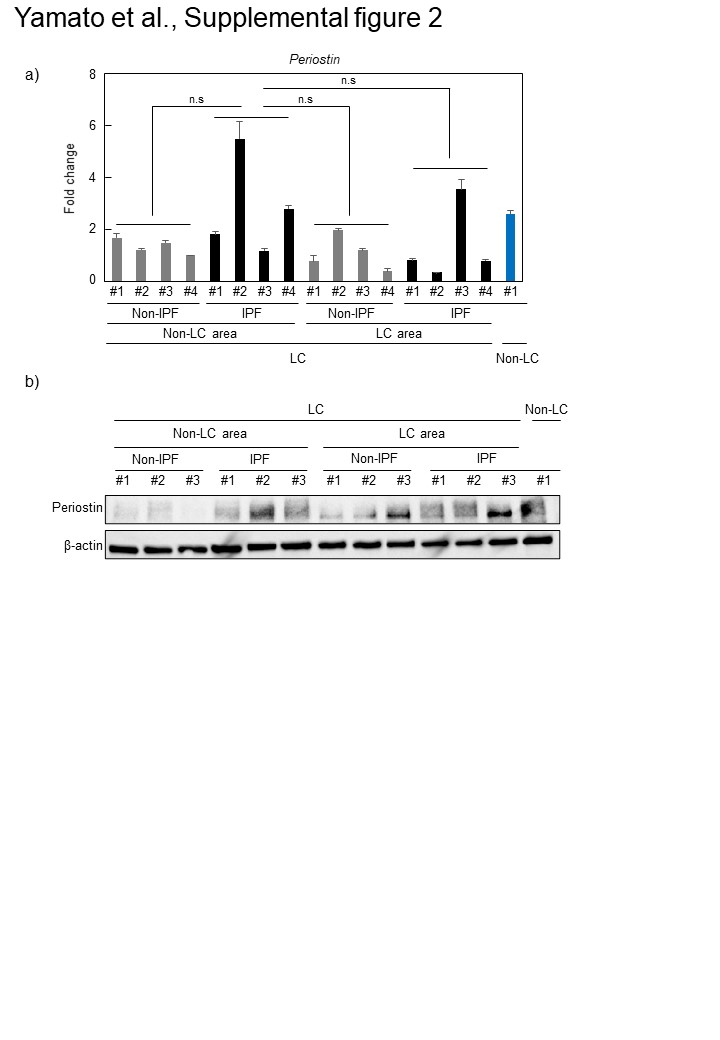
**

**Supplemental Figure 4. RT-PCR evaluation of the difference between NHLF and DIPF**

a–c) Expressions of *COL1a1* (a), *TGF-β2* (b) and *ACTA2* (c) in NHLF and DIPF cells were compared using RT-PCR. Statistical significance was tested with the Mann-Whitney U test. *P < 0.05, n.s., non- significant.

RT-PCR, real time-PCR; NHLF, normal human lung fibroblast; DIPF, disease human lung fibroblast-IPF; *COL1A1*, *collagen type I alpha 1 chain*; *TGF-β2*, *transforming growth factor β2*; *ACTA2*, *actin alpha 2*.

**

**

**Supplemental Figure 5. Expression of *ITB3*-depleted NSLCL cells**


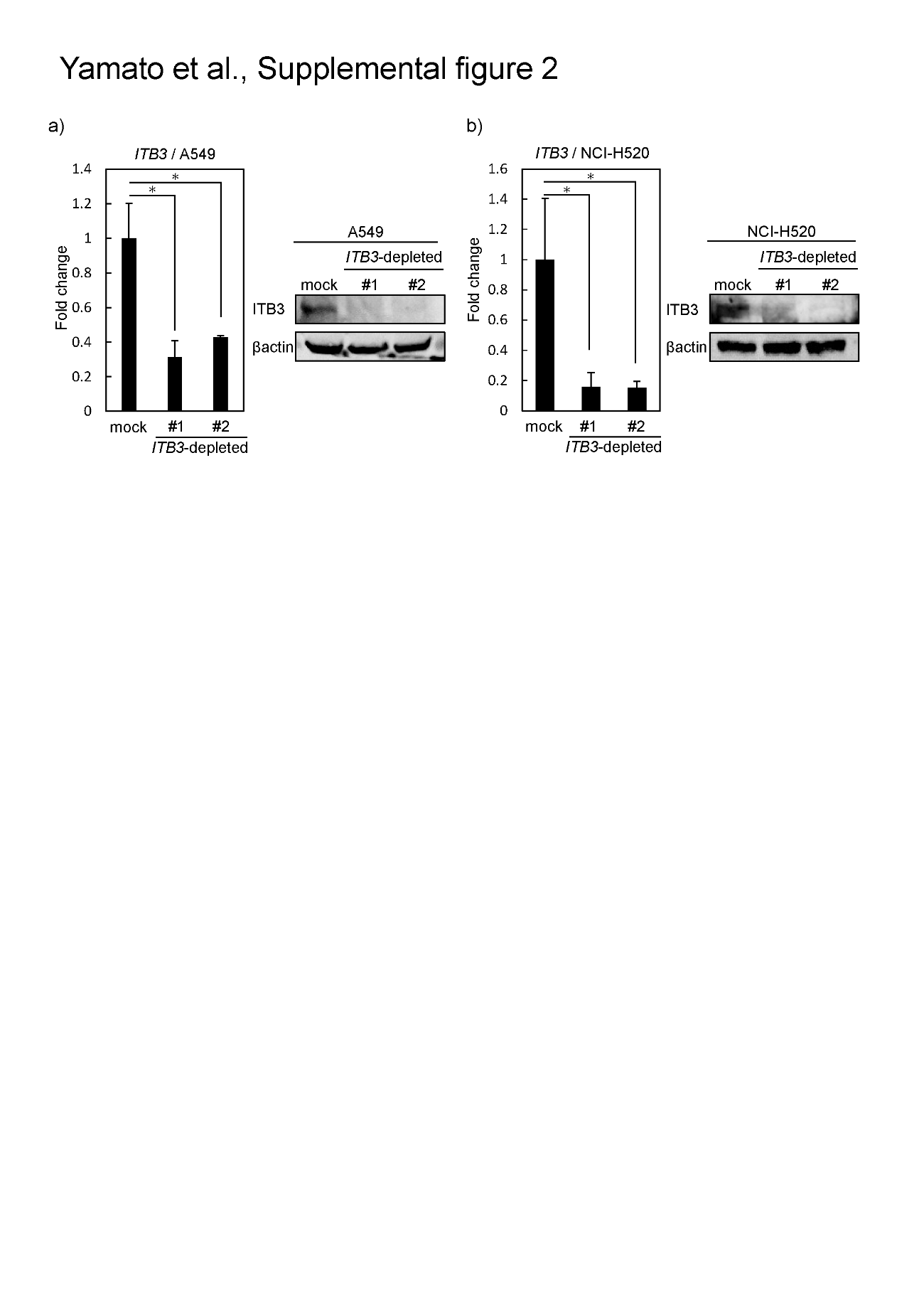
We generated A549 or NCI-H520 cells transfected with *ITB3* shRNA to constitutively reduce *ITB3* expression. a, b) Expression of *ITB3* was evaluated by RT-qPCR and WB in *ITB3*-depleted A549 (a) and NCI-H520 (b) cells. Mock A549 and NCI-H520 cells were used as controls. Statistical significance was tested with the Mann-Whitney U test. *P < 0.05. RT-PCR, real time-PCR; *ITB3*, *integrin β3*.


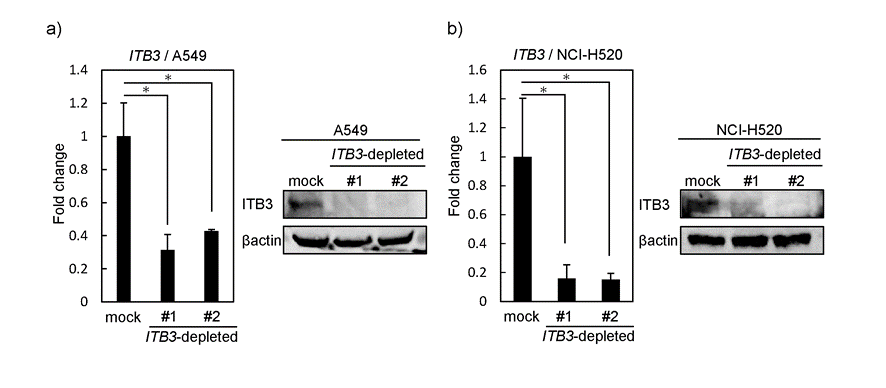


**Supplemental Figure 6. Effect of periostin receptor silencing on NSCLC cells *in vivo***

*ITB3*-depleted A549 or NCI-H520 cells were mixed with or without DIPF in a 5:1 ratio, co-injected subcutaneously into nude mice (n= 5), and sacrificed 28 days after co-injection. a, b) In *ITB3*-depleted A549 (NCI-H520) cells with or without DIPF co-injection, tumor size after co-injection of cells is plotted over time. *P < 0.05, **P < 0.01; n.s., non-significant. *ITB3*, *integrin β3*; DIPF, disease human lung fibroblast-IPF.

*
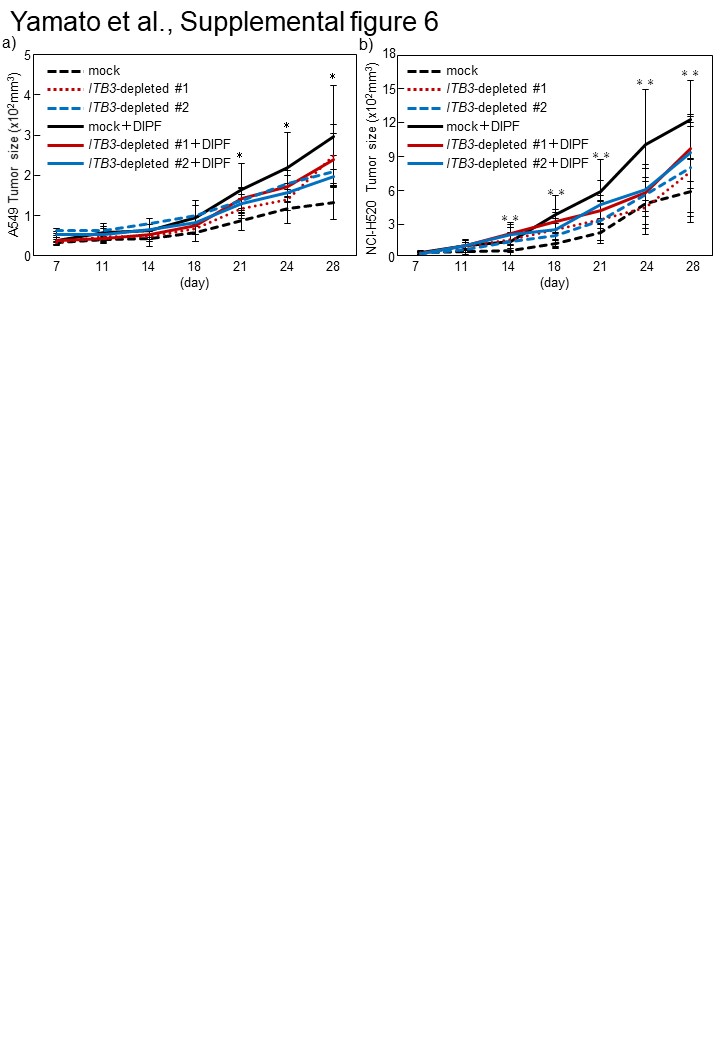
*

**Supplemental Figure 7. Expression of periostin in fibroblasts isolated from patient specimens.**

Fibroblasts were isolated from resected lung specimens obtained from IPF (IPFfs; n=7) and non-IPF (NLfs; n=5) patients, as well as from tumor tissues of IPF patients with lung cancer (LC) (IPF-CAFs; n=6). One patient in the IPFfs group had no evidence of LC. (a–d) mRNA expressions of POSTN (a), COL1a1 (b), TGF-β2 (c), and ACTA2 (d) were compared between the NLfs and IPFfs groups using RT-PCR. (e) The concentration of periostin in CM from the NLfs and IPFfs groups was determined by ELISA. (f) Periostin expression in IPFfs and IPF-CAFs from the same patients (n=6) was examined using RT-PCR. Significance was tested with a Mann-Whitney U test. **P <0.01. n.s., non-significant; IPF, idiopathic pulmonary fibrosis; IPFfs, IPF lung-derived fibroblasts; NLfs, normal lung-derived fibroblasts; LC, lung cancer; CAFs, cancer-associated fibroblasts; POSTN, periostin; COL1A1, collagen type I alpha 1 chain; TGF- β2, transforming growth factor β2; ACTA2, actin alpha 2; RT-PCR, real-time PCR; CM, conditioned media; ELISA, enzyme-linked immunosorbent assay
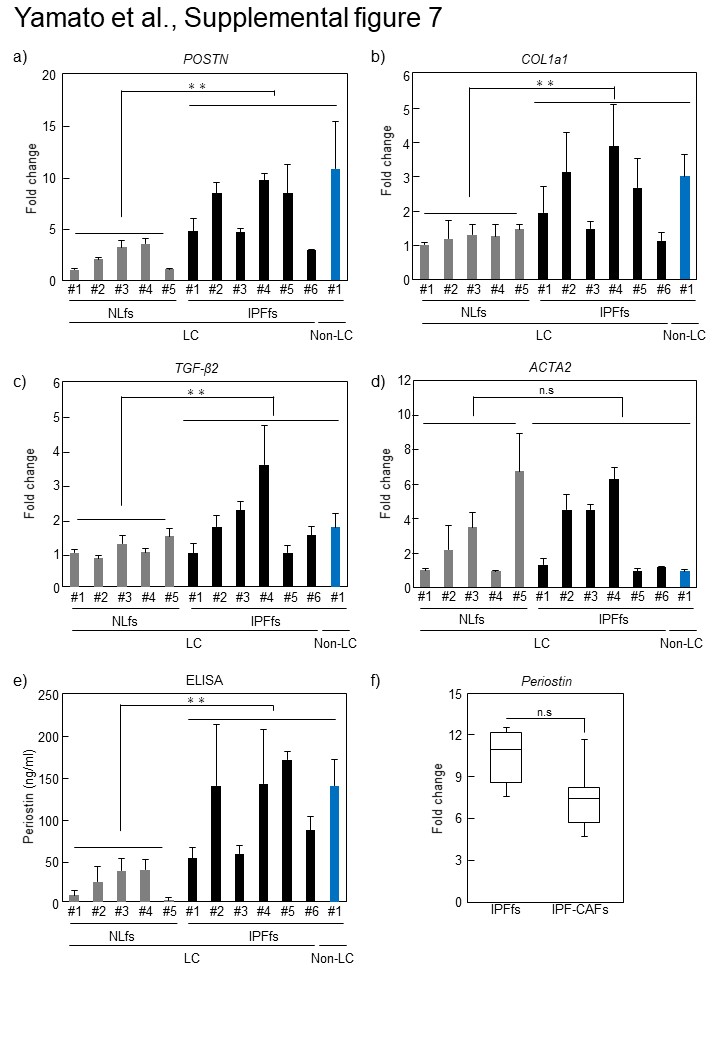


**Supplemental Table 1. Clinicopathological characteristics of patients with resected lung cancer.**

| Factors | LC-non-PF n=512 (95.2%) | LC-IPF n=26 (4.8%) | P-value |
| --- | --- | --- | --- |
| Age (mean, range) | 69±37 | 73±10 | <0.01 |
| Sex (M/F) | 301/211 | 21/5 | 0.02 |
| Brinkman index (≦600/>600) | 286/226 | 6/20 | 0.02 |
| %VC (%) (mean, range) | 100.9±14.5 | 97.2±18.5 | 0.66 |
| FEV1.0% (%) (mean, range) | 74.1±9.7 | 74.4±8.2 | 0.92 |
| DLco (ml/min/mmHg) (mean, range) | 14.8±4.8 | 11.0±3.2 | <0.01 |
| CEA (ng/ml) (≦3.1/>3.1) | 211/301 | 5/21 | 0.82 |
| CYFRA (ng/ml) (≦3.5/>3.5) | 460/52 | 17/8 | 0.02 |
| PET-CT SUVmax (mean, range) (n=353) | 3.5 (0.6-28.6) | 5.9 (1.5-31.7) | 0.02 |
| Histological type (Ad/Sq/Other) | 413/85/14 | 11/12/3 | <0.01 |
| Tumor size (cm) (≦2.0/>2.0) | 221/291 | 2/23 | <0.01 |
| Pathological stage (I/II,III,IV) | 416/96 | 13/13 | 0.06 |

LC-non-IPF, lung cancer patients without idiopathic pulmonary fibrosis; LC-IPF, lung cancer patients with idiopathic pulmonary fibrosis; %VC, percentage predicted vital capacity; FEV1.0%, percentage predicted forced expiratory volume in 1 second; DLco, diffusing capacity for carbon monoxide; CEA, carcinoembryonic antigen;

CYFRA, cytokeratin fragment; PET-CT, positron emission tomography-computed tomography; SUVmax, maximum standardized uptake value.

**Supplemental Table 2. Clinicopathological characteristics of patients whose frozen specimens were used for western blot in supplemental figure 1a and 3b.**

| Factors | Non-IPF (n=3) | IPF (n=4) | P-value |
| --- | --- | --- | --- |
| Age (mean, range) | 73±1.4 | 73.0±6.7 | 0.76 |
| Sex (M/F) | 2/1 | 3/1 | 0.26 |
| Brinkman index (≦600/>600) | 0/3 | 1/3 | 0.22 |
| %VC (%) (mean, range) | 101.6±20.3 | 100.4±14.5 | 0.94 |
| FEV1.0% (%) (mean, range) | 71.6±4.3 | 74.0±1.7 | 0.87 |
| DLco (ml/min/mmHg) (mean, range) | 17.7±1.6 | 11.5±2.2 | 0.01 |
| Lung cancer ( with/without) | 3/0 | 3/1 |  |
| CEA (ng/ml) (≦3.1/>3.1) | 2/1 | 1/3 | 0.66 |
| CYFRA (ng/ml) (≦3.5/>3.5) | 3/0 | 4/0 |  |
| PET-CT SUVmax (mean, range) (n=2) | 9.5±0.9 | － |  |
| Histological type (Ad/Sq/Other) | 1/2/0 | 1/2/0 | 0.54 |
| Tumor size (cm) (≦2.0/>2.0) | 1/2 | 0/3 | 0.24 |
| Pathologic Stage (I/II/III/IV) | 2/1/0/0 | 2/1/0/0 | 0.74 |
| Outcome |  |  |  |
| Alive | 3 | 2 | 0.26 |
| Alive with cancer | 0 | 1 | 0.26 |
| Dead of cancer | 0 | 1 | 0.26 |
| Dead of respiratory failure | 0 | 0 |  |

Non-IPF, Non- idiopathic pulmonary fibrosis; IPF, idiopathic pulmonary fibrosis;

%VC: % vital capacity; FEV1.0%, forced expiratory volume % in first second; DLco, diffusing capacity of the lung for carbon monoxide; CEA, carcinoembryonic antigen; CYFRA, cytokeratin fragment; PET, positron emission tomography; SUVmax, standardized uptake value max; *, One patient without lung cancer was included in the IPF group.

**Supplemental Table 3. Clinicopathological characteristics of patients whose frozen specimens were used for RT-PCR in supplemental figure 3a.**

| Factors | Non-IPF (n=4) | IPF (n=5) | P-value |
| --- | --- | --- | --- |
| Age (mean, range) | 74±2.1 | 72.8±6.9 | 0.95 |
| Sex (M/F) | 3/1 | 4/1 | 0.85 |
| Brinkman index (≦600/>600) | 0/4 | 3/2 | 0.26 |
| %VC (%) (mean, range) | 92.2±24.0 | 10.2±13.6 | 0.62 |
| FEV1.0% (%) (mean, range) | 69.1±5.6 | 76.2±4.8 | 0.12 |
| DLco (ml/min/mmHg) (mean, range) | 15.0±4.9 | 12.5±2.8 | 0.29 |
| Lung cancer (with/without) | 4/0 | 4/1 |  |
| CEA (ng/ml) (≦3.1/>3.1) | 2/2 | 2/3 | 0.76 |
| CYFRA (ng/ml) (≦3.5/>3.5) | 3/1 | 5/0 | 0.18 |
| PET-CT SUVmax (mean, range) (n=4) | 10.7±1.8 | － |  |
| Histological type (Ad/Sq/Other) | 1/3/0 | 1/3/0 | 0.53 |
| Tumor size (cm) (≦2.0/>2.0) | 1/3 | 0/4 | 0.26 |
| Pathological stage (I/II/III/IV) | 3/1/0/0 | 3/1/0/0 | 0.58 |
| Outcome |  |  |  |
| Alive | 4 | 3 | 0.15 |
| Alive with cancer | 0 | 1 | 0.25 |
| Dead of cancer | 0 | 1 | 0.25 |
| Dead of respiratory failure | 0 | 0 |  |

Non-IPF, Non- idiopathic pulmonary fibrosis; IPF, idiopathic pulmonary fibrosis;

%VC: % vital capacity; FEV1.0%, forced expiratory volume % in first second; DLco, diffusing capacity of the lung for carbon monoxide; CEA, carcinoembryonic antigen; CYFRA, cytokeratin fragment; PET, positron emission tomography; SUVmax, standardized uptake value max; *, One patient without lung cancer was included in the IPF group.

**Supplemental Table 4. Clinicopathological characteristics of patients with fibroblasts isolated from resected specimens.**

| Factors | NLfs (n=5) | IPFfs (n=7) | P-value |
| --- | --- | --- | --- |
| Age (mean, range) | 67±13 | 75±5 | 0.11 |
| Sex (M/F) | 4/1 | 6/1 | 0.79 |
| Brinkman index (≦600/>600) | 2/3 | 1/6 | 0.11 |
| %VC (%) (mean, range) | 91.9±8.9 | 106.3±21.6 | 0.14 |
| FEV1.0% (%) (mean, range) | 80.7±11.5 | 72.5±8.0 | 0.14 |
| DLco (ml/min/mmHg) (mean, range) | 15.7±2.9 | 11.0±2.6 | <0.01 |
| Lung cancer (with/without) | 5/0 | 6/1 |  |
| CEA (ng/ml) (≦3.1/>3.1) | 4/1 | 1/5 | 0.07 |
| CYFRA (ng/ml) (≦3.5/>3.5) | 4/1 | 6/0 | 0.75 |
| PET-CT SUVmax (mean, range) (n=11) | 10.4±10.9 | 12.5±8.1 | 0.69 |
| Histological type (Ad/Sq/Other) | 3/2/0 | 1/4/1 | 0.26 |
| Tumor size (cm) (≦2.0/>2.0) | 2/3 | 0/6 | <0.01 |
| Pathological stage (I/II/III/IV) | 5/0/0/0 | 2/2/1/1 | 0.57 |
| Outcome |  |  |  |
| Alive | 3 | 3 | 0.15 |
| Alive with cancer | 1 | 2 | 0.04 |
| Dead of cancer | 0 | 2 | 0.15 |
| Dead of respiratory failure | 1 | 0 | 0.19 |

NLFs, normal lung-derived fibroblast; IPFfs, idiopathic pulmonary firosis-derived fibroblast; %VC, percentage predicted vital capacity; FEV1.0%, percentage predicted forced expiratory volume in 1 second; DLco, diffusing capacity of the lung for carbon monoxide; CEA, carcinoembryonic antigen; CYFRA, cytokeratin fragment; PET-CT, positron emission tomography-computed tomography; SUVmax, maximum standardized uptake value. *, One patient without lung cancer was included in the IPFfs group.
