# Supplementary material for: Periostin secreted by activated fibroblasts in idiopathic pulmonary fibrosis promotes tumorigenesis of non-small cell lung cancer
Source: Sci Rep. 2021 Oct 26;11:21114. doi: 10.1038/s41598-021-00717-5 (PMC8548404; doi:10.1038/s41598-021-00717-5)
Supplement: Supplementary file 2 — Supplementary Information 2. [file 41598_2021_717_MOESM2_ESM.docx]

Periostin secreted by activated fibroblasts in idiopathic pulmonary fibrosis promotes tumorigenesis of non-small cell lung cancer

**Authors**

Hiroyuki Yamato, Kenji Kimura, Eriko Fukui, Takashi Kanou, Naoko Ose, Soichiro Funaki, Masato Minami, and Yasushi Shintani

**Full-length gels and blots**

Fig. 1f


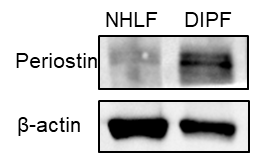


The membrane was cut prior to hybridization with antibodies. And these blots were returned to the same position as before the cut and then visualized. The line where the gel was cut is indicated by the blue line.

Periostin (Fig. 1f)


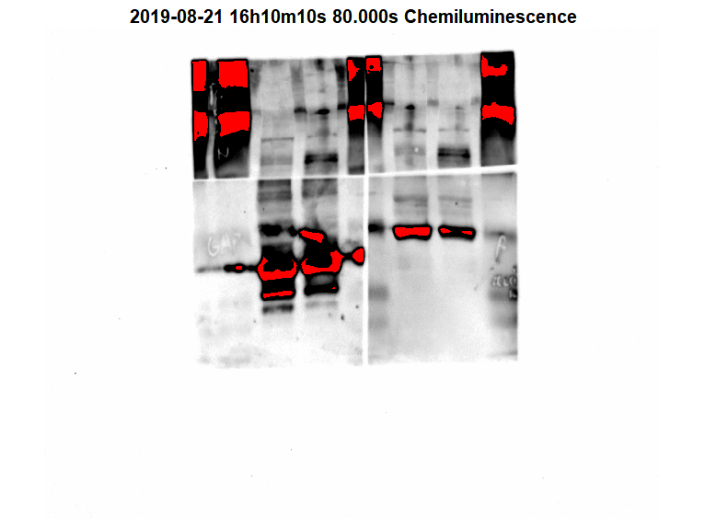


GAPDH

Periostin

**Periostin**

Beta-actin

Beta-actin (Fig. 1f)


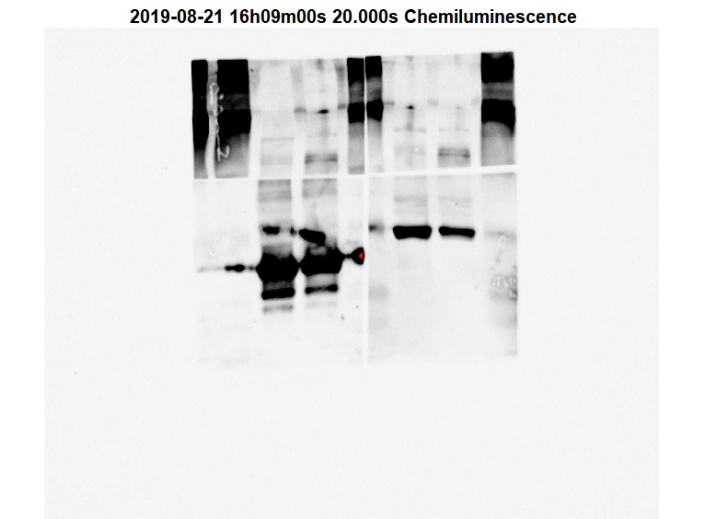


**Beta-actin**

Multiple exposure images (Fig. 1f)


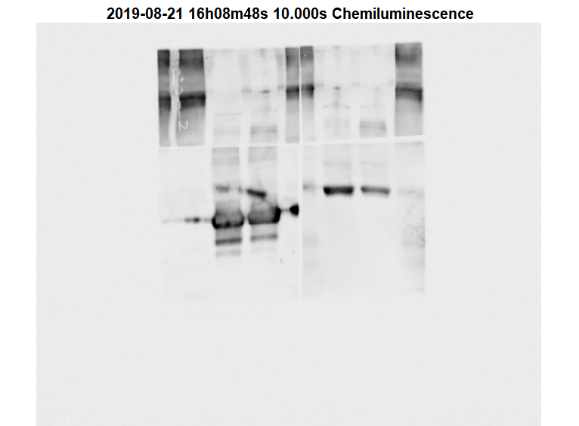

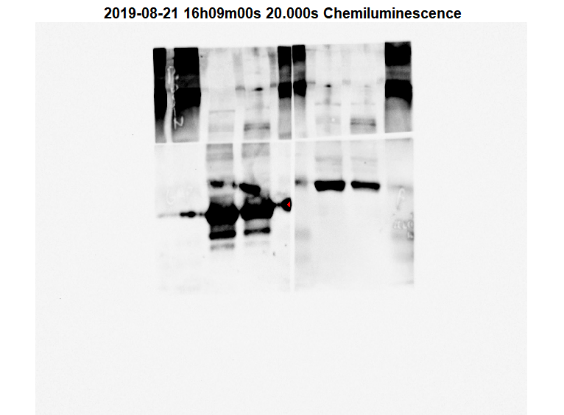

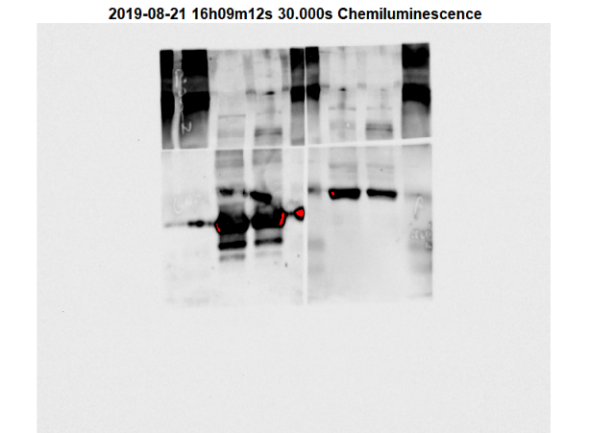

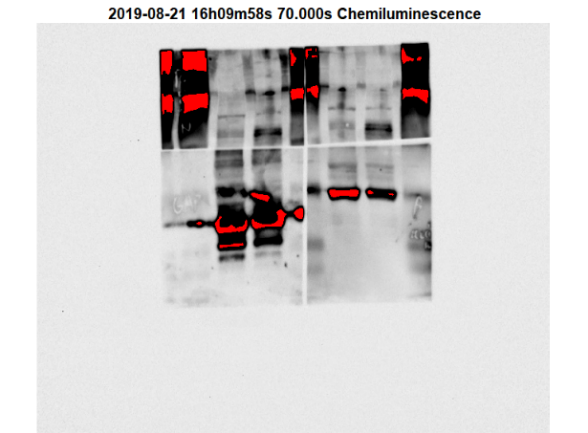

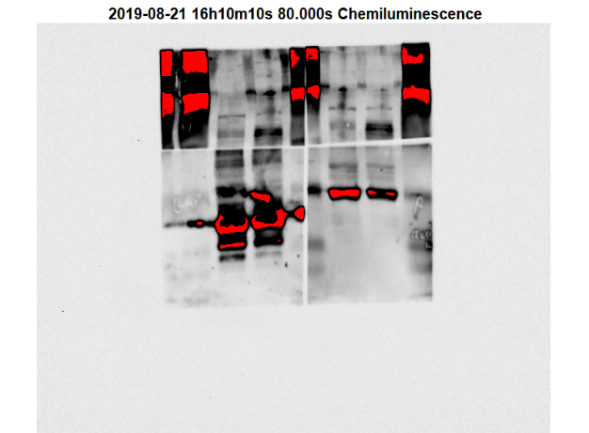


Fig. 2c

pErk

tErk


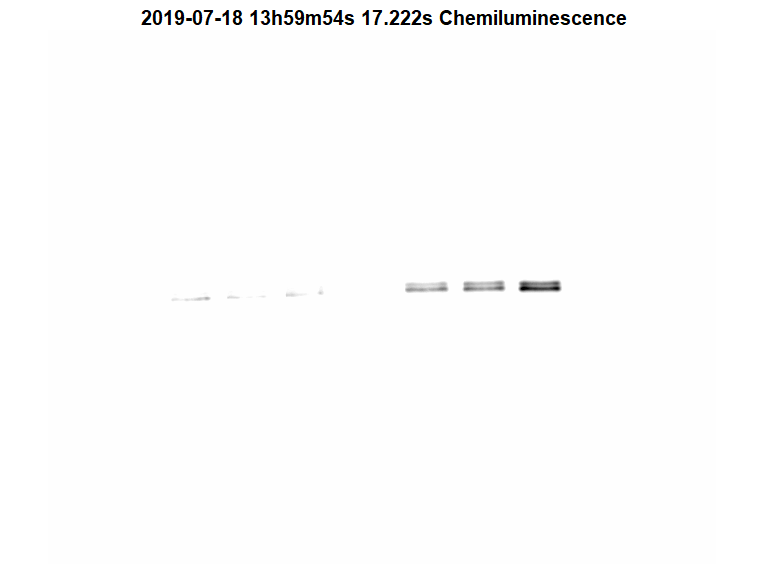

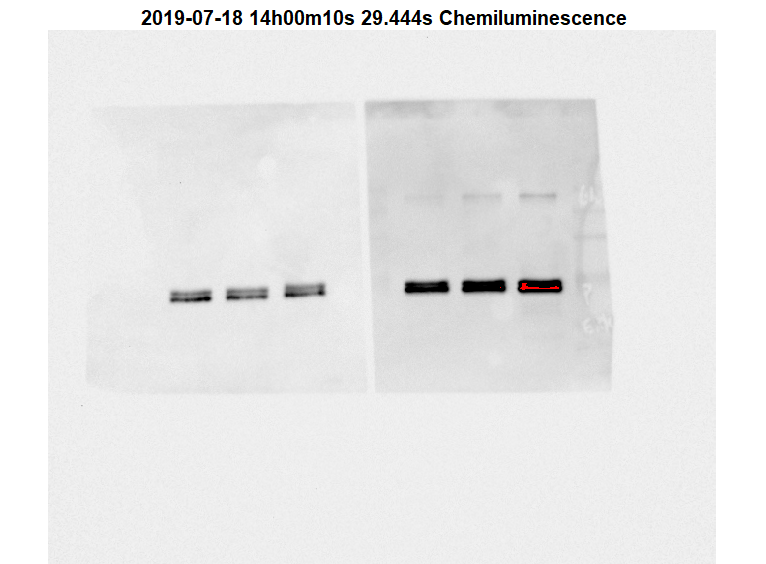


pAkt

tAkt


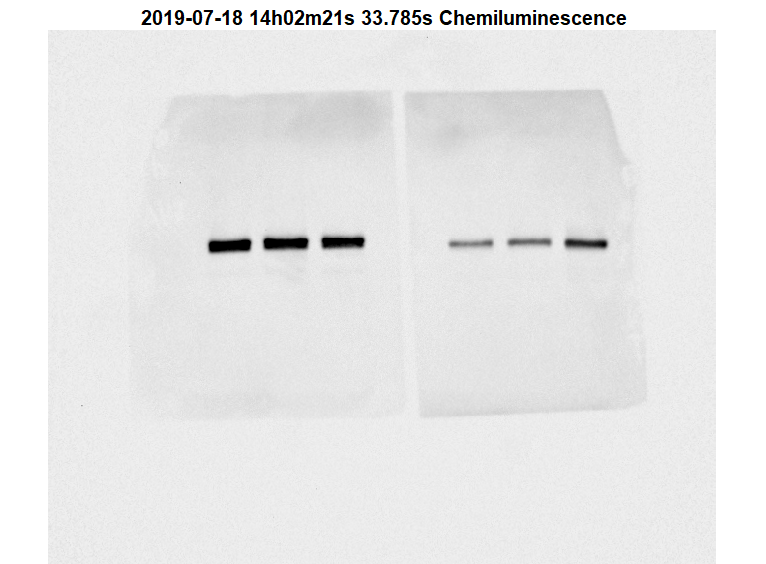

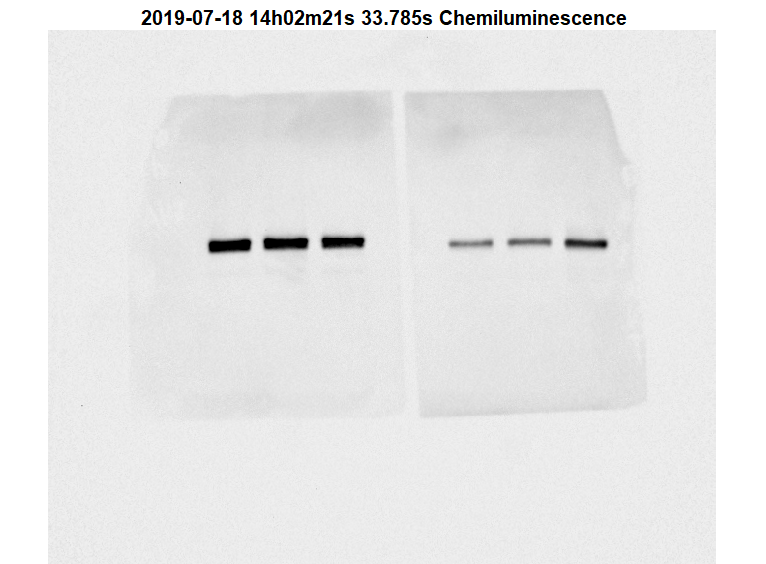


nCM

iCM

Control

A549

pErk (Fig. 2c)


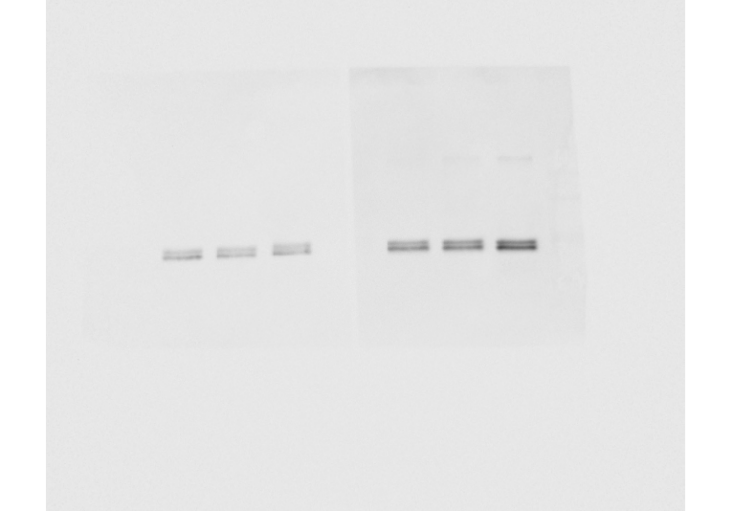


**pErk**

tErk (Fig. 2c)


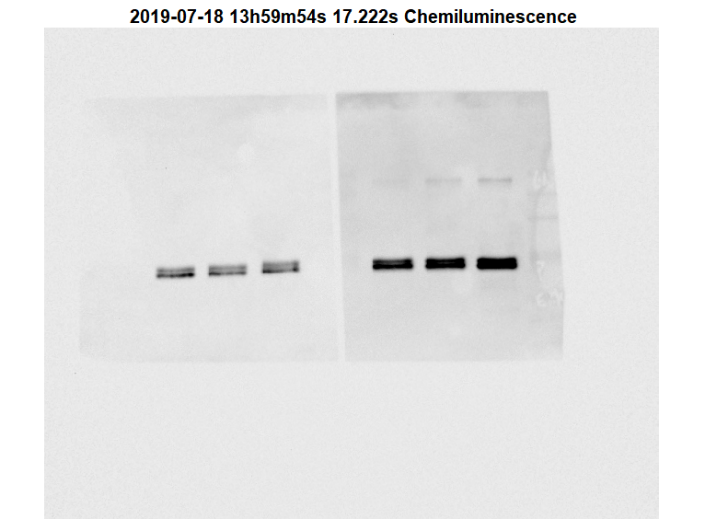


**tErk**

pAkt/tAkt (Fig. 2c)


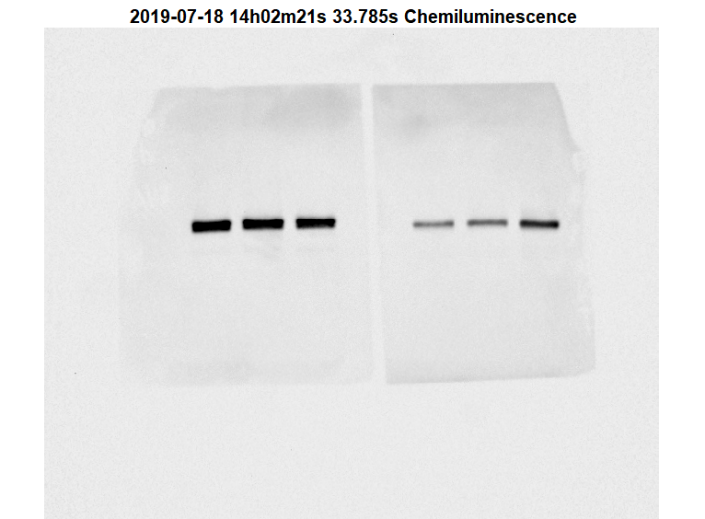


**pAkt**

**tAkt**

Fig. 2d


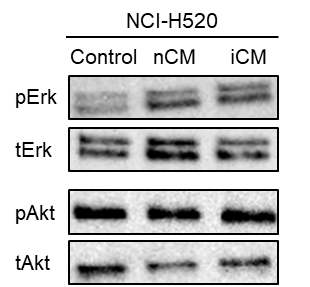


The membrane was cut prior to hybridization with antibodies. The top membranes were used for pAkt and tAkt, and the bottom membranes were used for pErk and tErk. Then, pErk and tErk, pAkt and tAkt were aligned up and down for visualization. The reason for this was to check the loading in one and also to make sure that each set does not shift.

pErk/tErk (Fig. 2d)


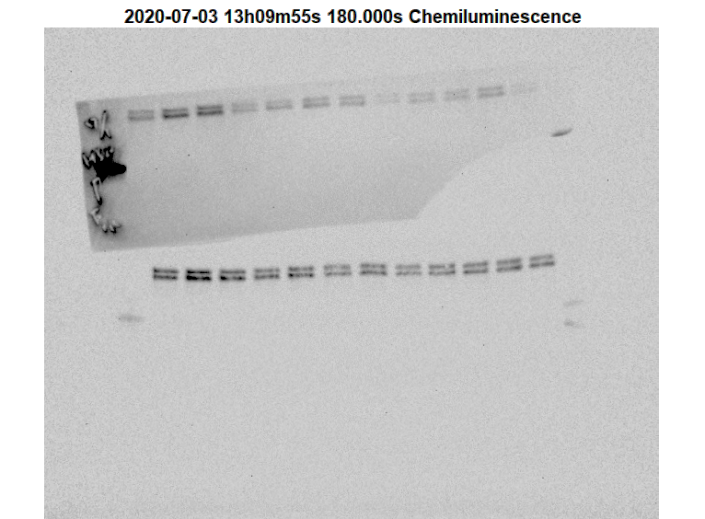


**tErk**

**pErk**

pAkt/tAkt (Fig. 2d)


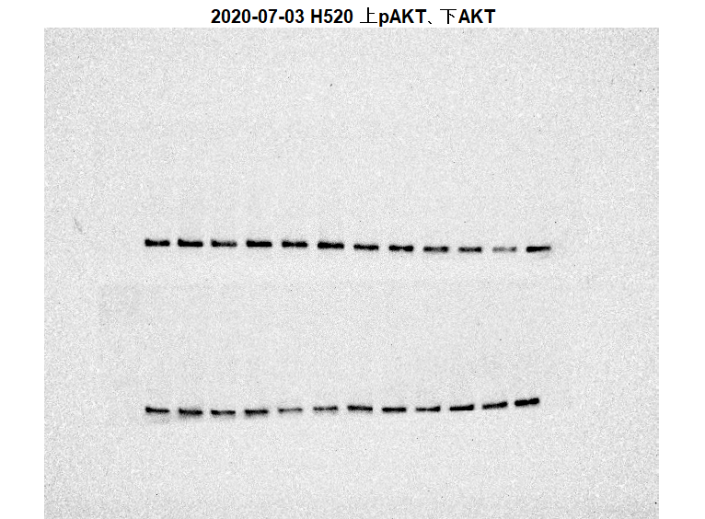


**tAkt**

**pAkt**

Membrane edges (tErk) are not visible, so we have shown full-length membranes (Fig. 2d).


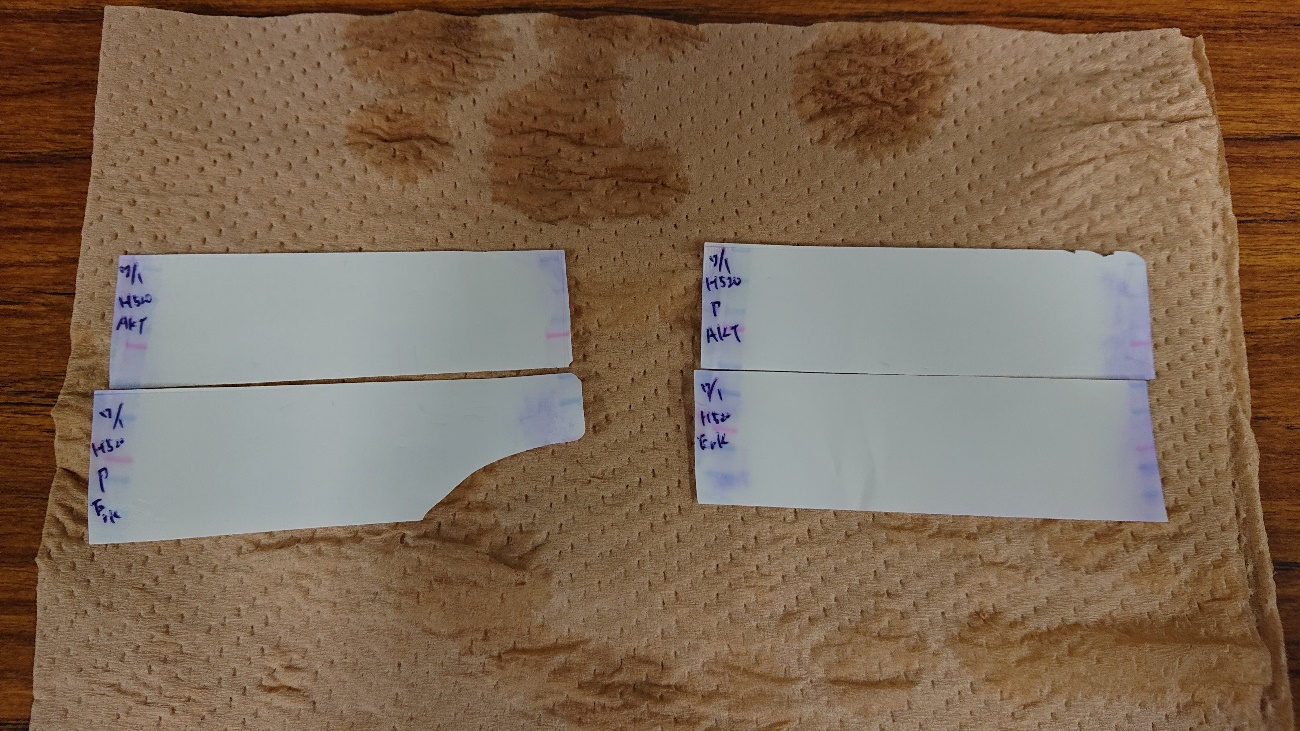


pErk

tErk

pAkt

tAkt

Multiple exposure images for pErk and tErk (Fig. 2d)


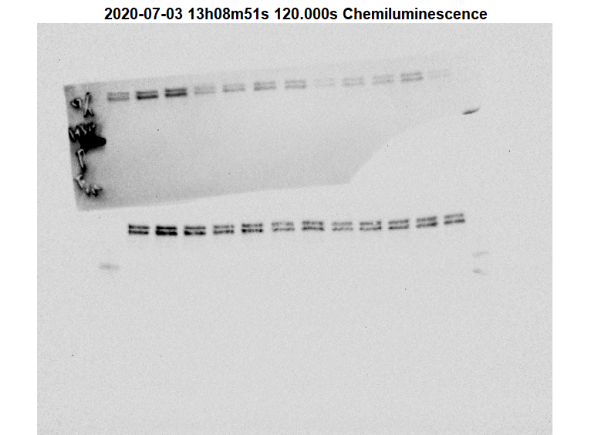

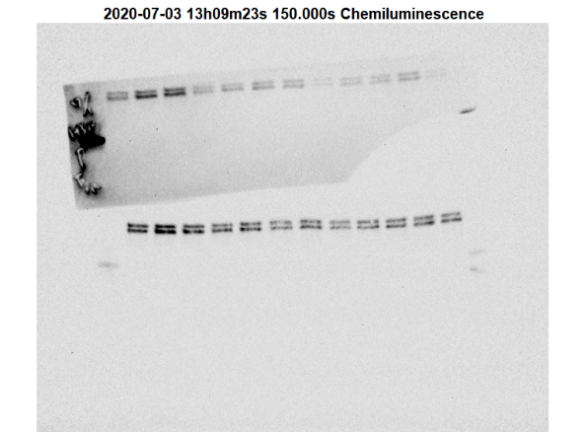


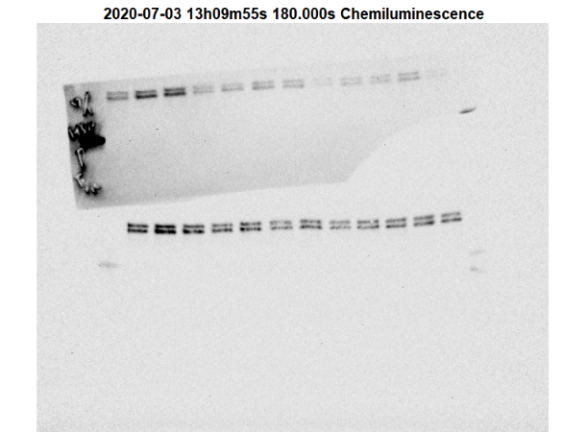

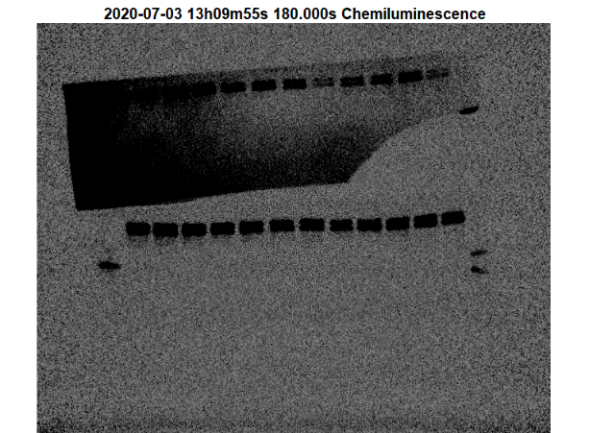


The blots placement during exposure for pErk/tErk (Fig. 2d)


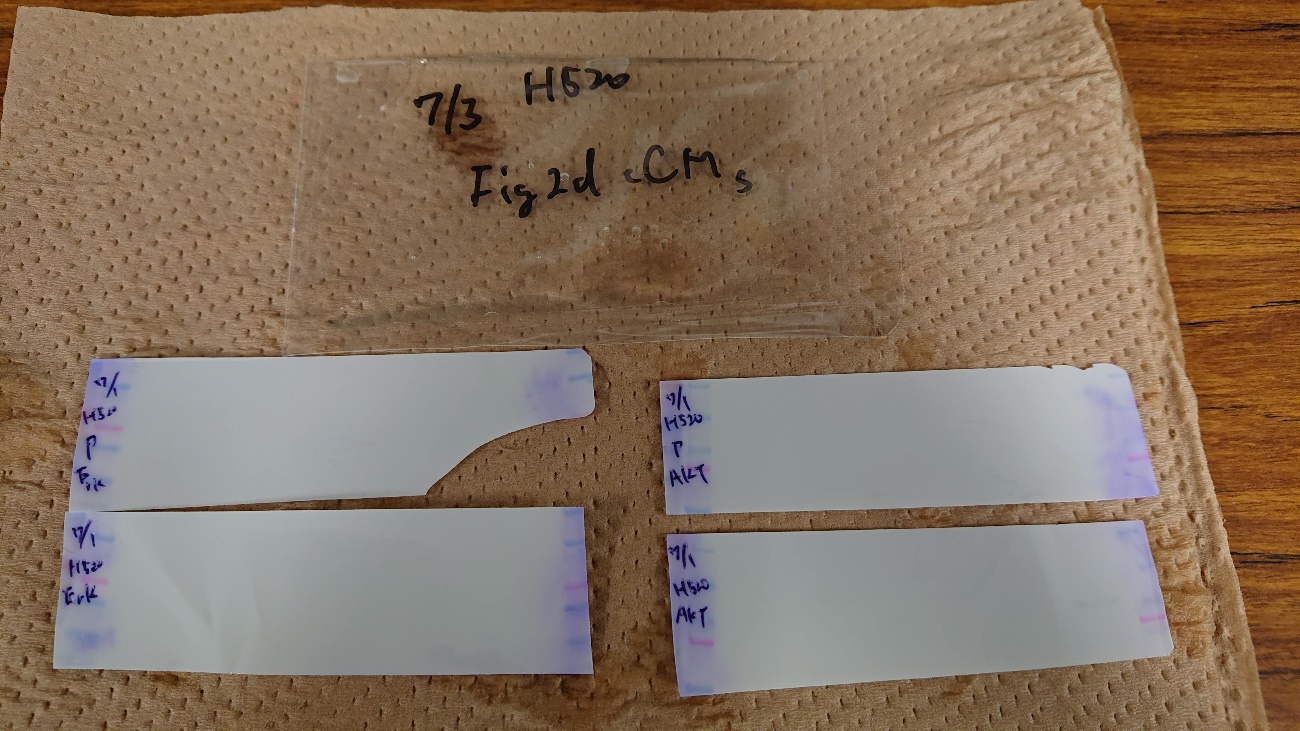


pErk

tErk

Fig. 2g


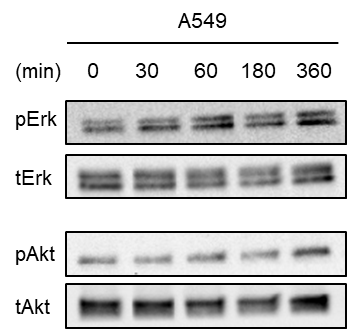


The membrane was cut prior to hybridization with antibodies. The top membranes were used for pAkt and tAkt, and the bottom membranes were used for pErk and tErk. Then, pErk and tErk, pAkt and tAkt were aligned up and down for visualization. The reason for this was to check the loading in one and also to make sure that each set does not shift.

pErk (Fig. 2g)


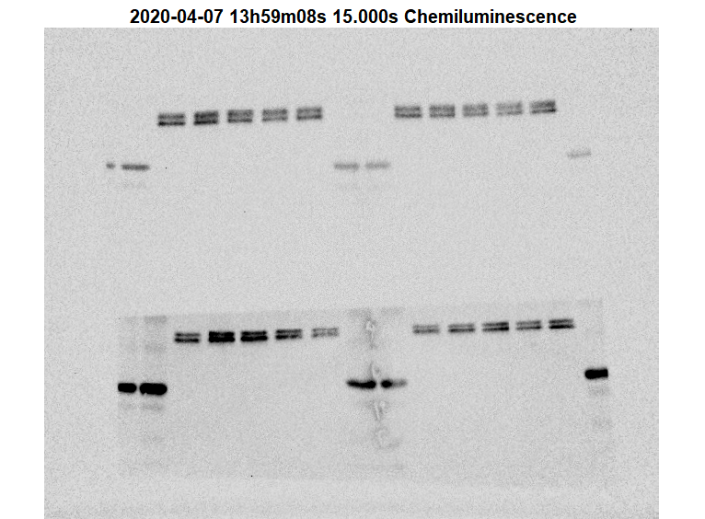


tErk

**pErk**

tErk (Fig. 2g)


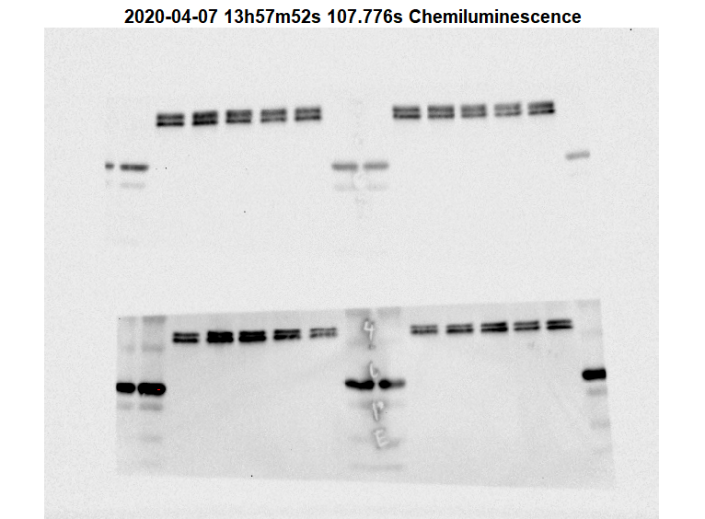


**tErk**

pErk

Multiple exposure images for tErk and pErk (Fig. 2g)


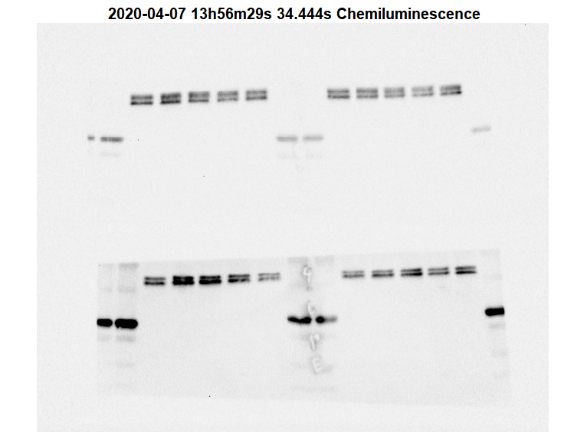

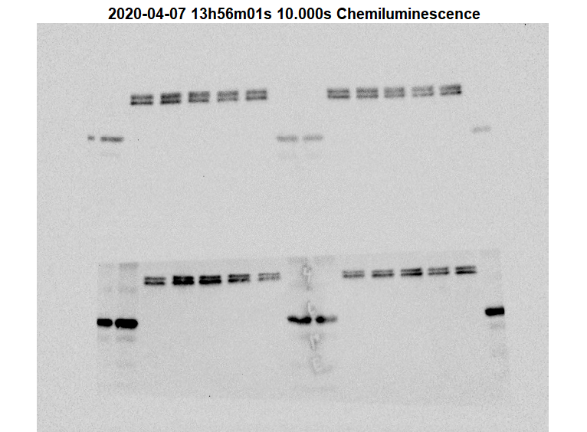

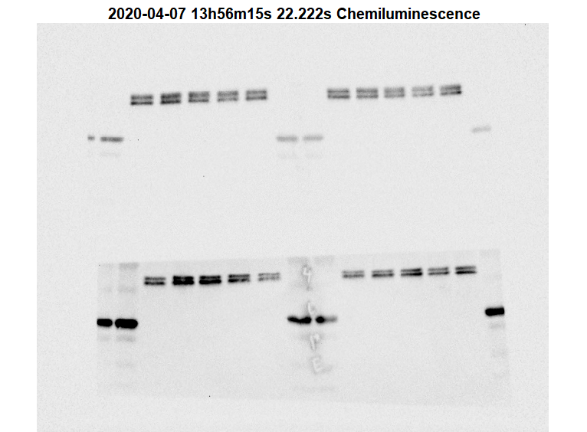

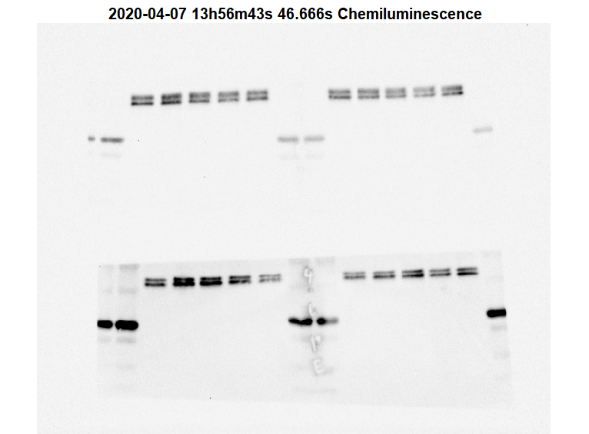


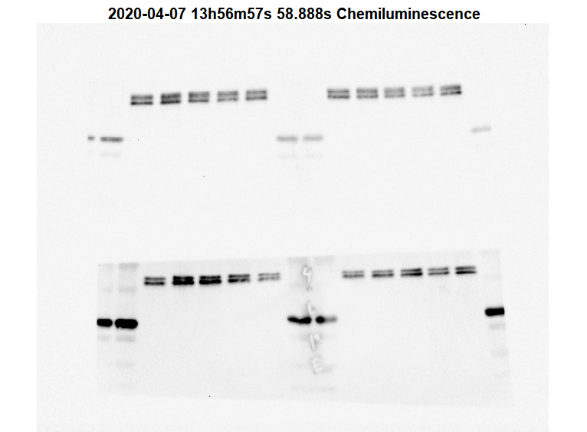

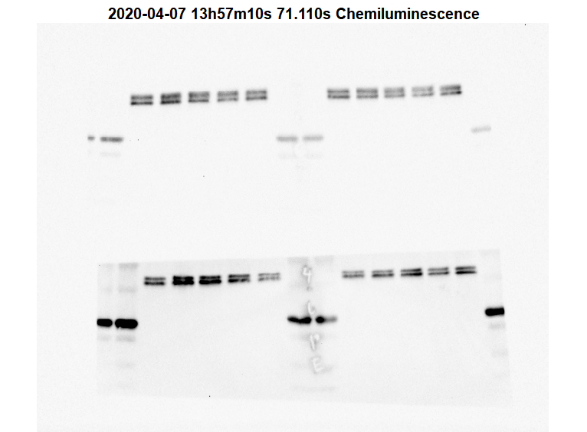

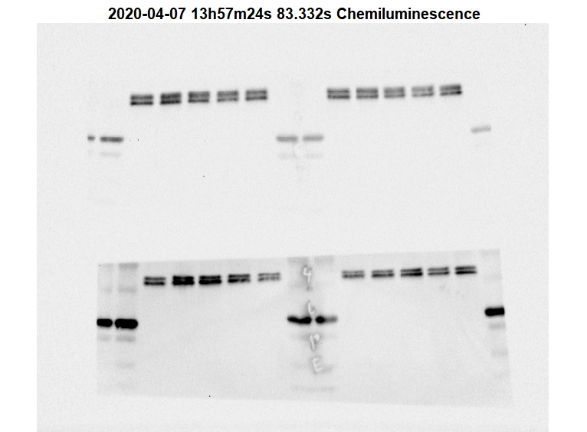

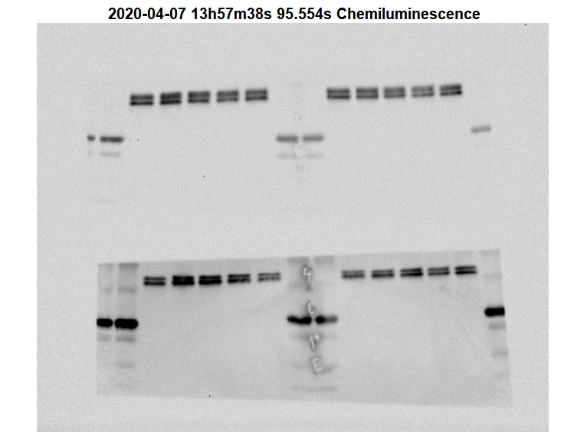

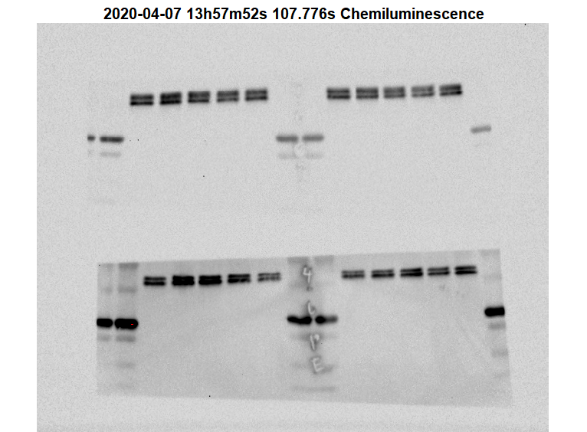

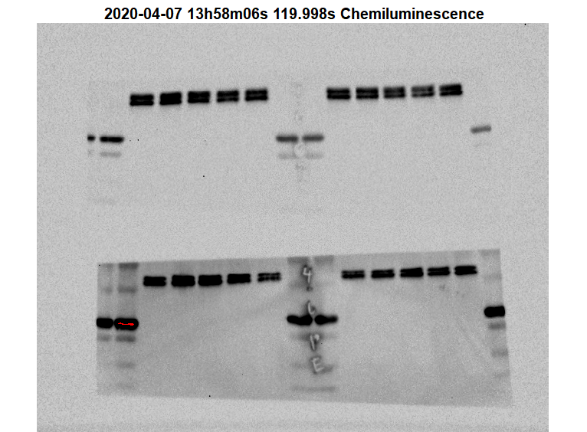

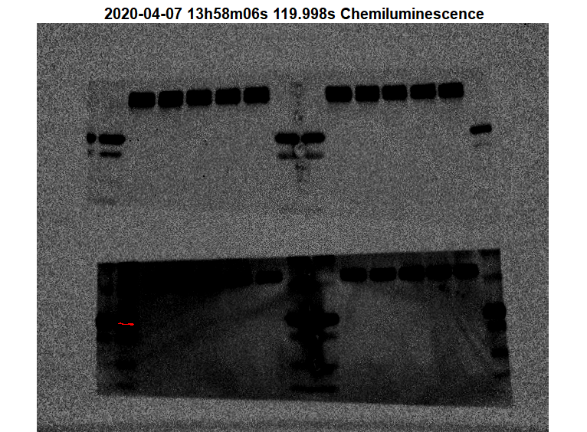


tAkt (Fig. 2g)


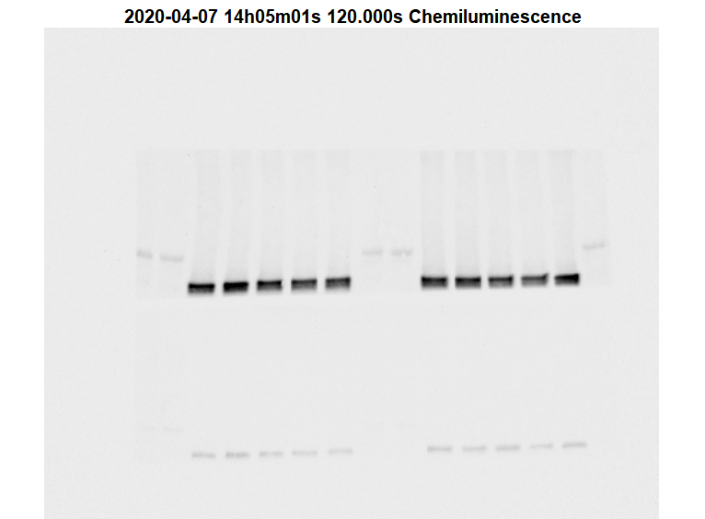


**tAkt**

pAkt

pAkt (Fig. 2g)

In the image shown below, pAkt was not visualized very well, so membrane for pAkt was exposed longer as shown below.


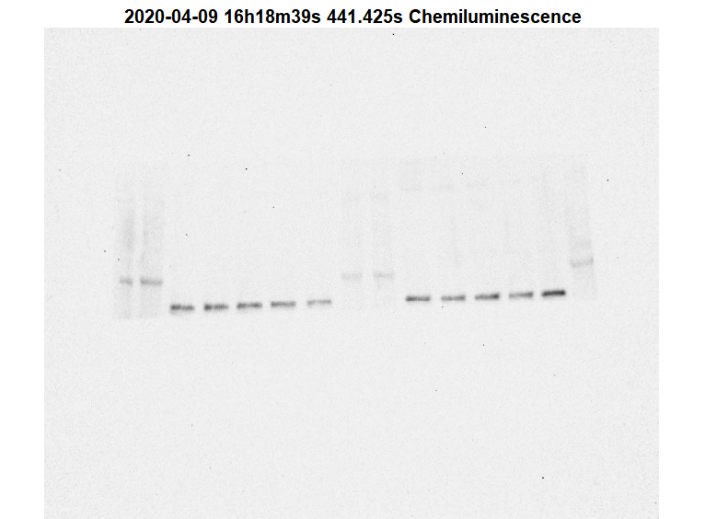


**pAkt**

Fig. 2h


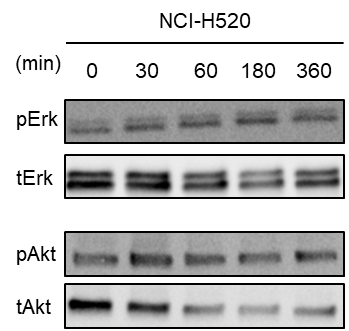


The membrane was cut prior to hybridization with antibodies. The top membranes were used for pAkt and tAkt, and the bottom membranes were used for pErk and tErk. The band on the far right which was indicated for 600 min stimulation was not necessary for this figure, so it has been omitted.

pErk (Fig. 2h)


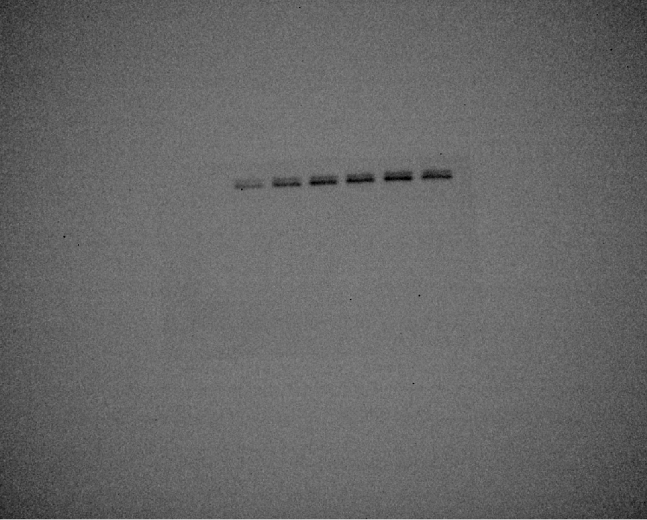


**pErk**

tErk (Fig. 2g)

**tErk**

The two images below are the same one with a different contrast, and the upper one was used for the figure.


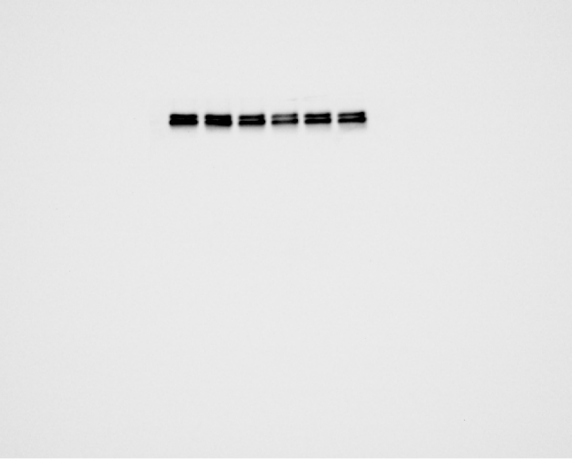

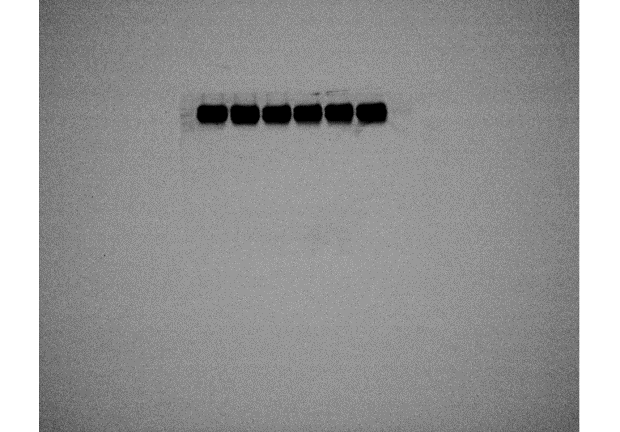


pAkt (Fig. 2g)


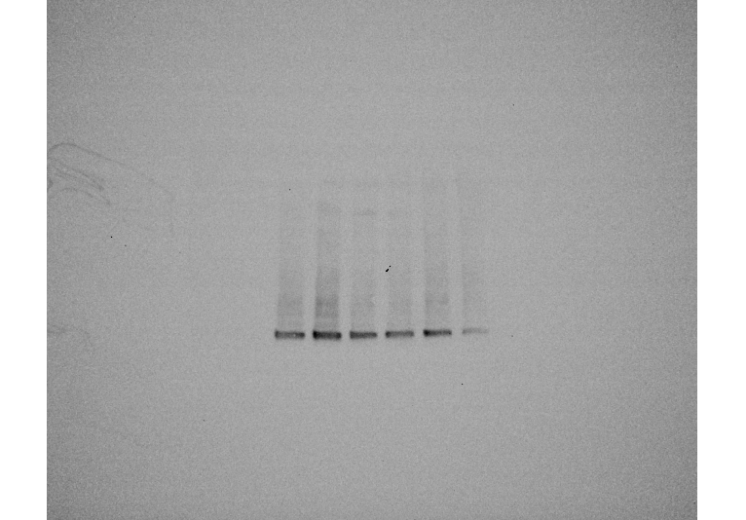


**pAkt**

tAkt (Fig. 2g)

The two images below are the same one with a different contrast, and the upper one was used for Figure.


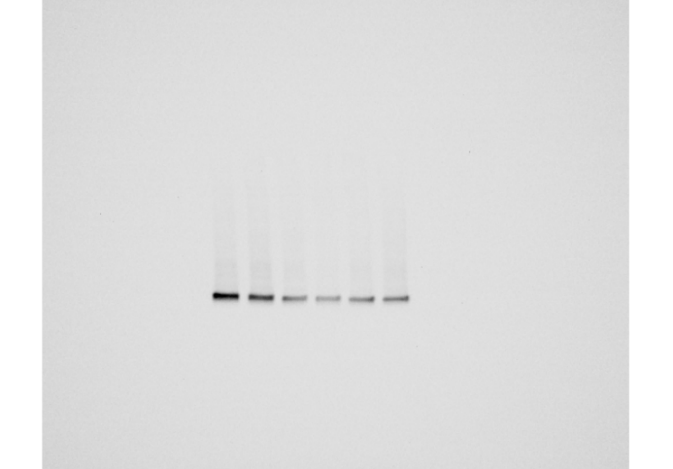

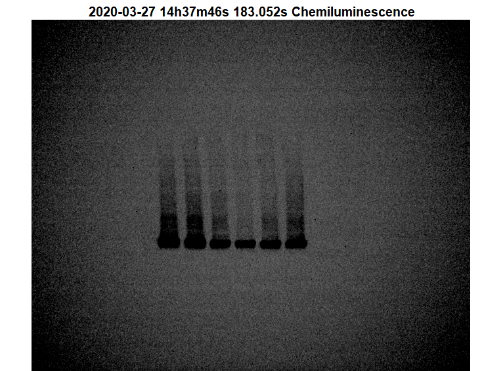


**tAkt**

Fig. 4e


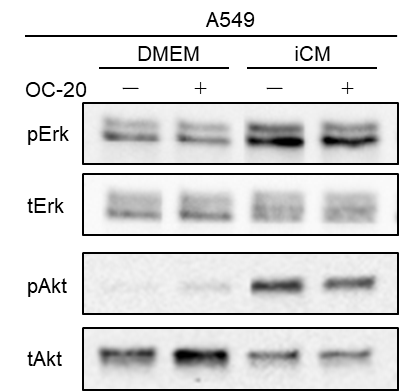


The membrane was cut prior to hybridization with antibodies. The top membranes were used for pAkt and tAkt, and the bottom membranes were used for pErk and tErk. Then, pErk and tErk, pAkt and tAkt were aligned up and down for visualization. The reason for this was to check the loading in one and also to make sure that each set does not shift.

pErk (Fig. 4e)


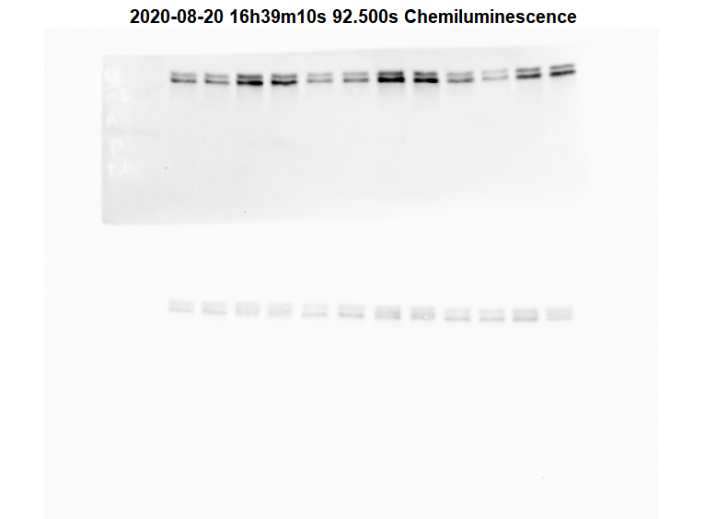


**pErk**

tErk (Fig. 4e)

In the image above, tErk was not visualized very well, so only tAkt was exposed longer again.


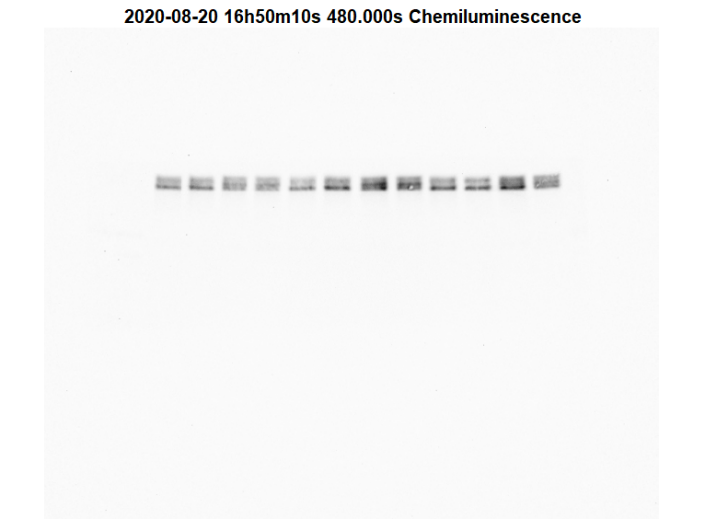


**tErk**

pAkt/ tAkt (Fig. 4e)


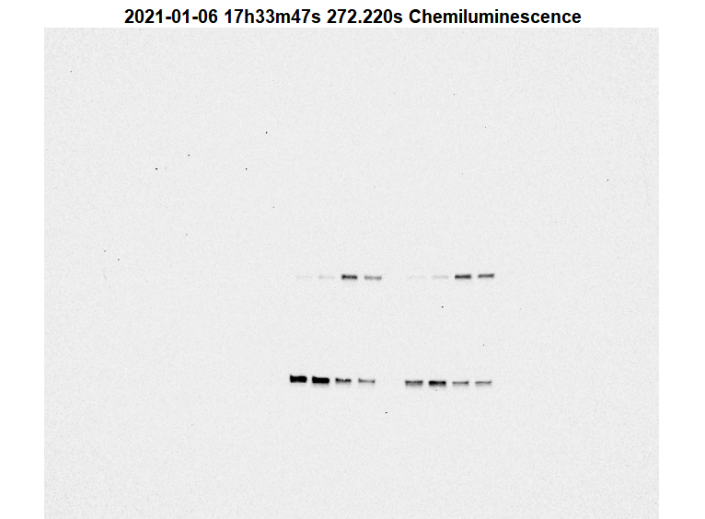


**pAkt**

**tAkt**

**Membrane edges (pAkt and tAkt) are not visible, so we have shown full-length membranes.**


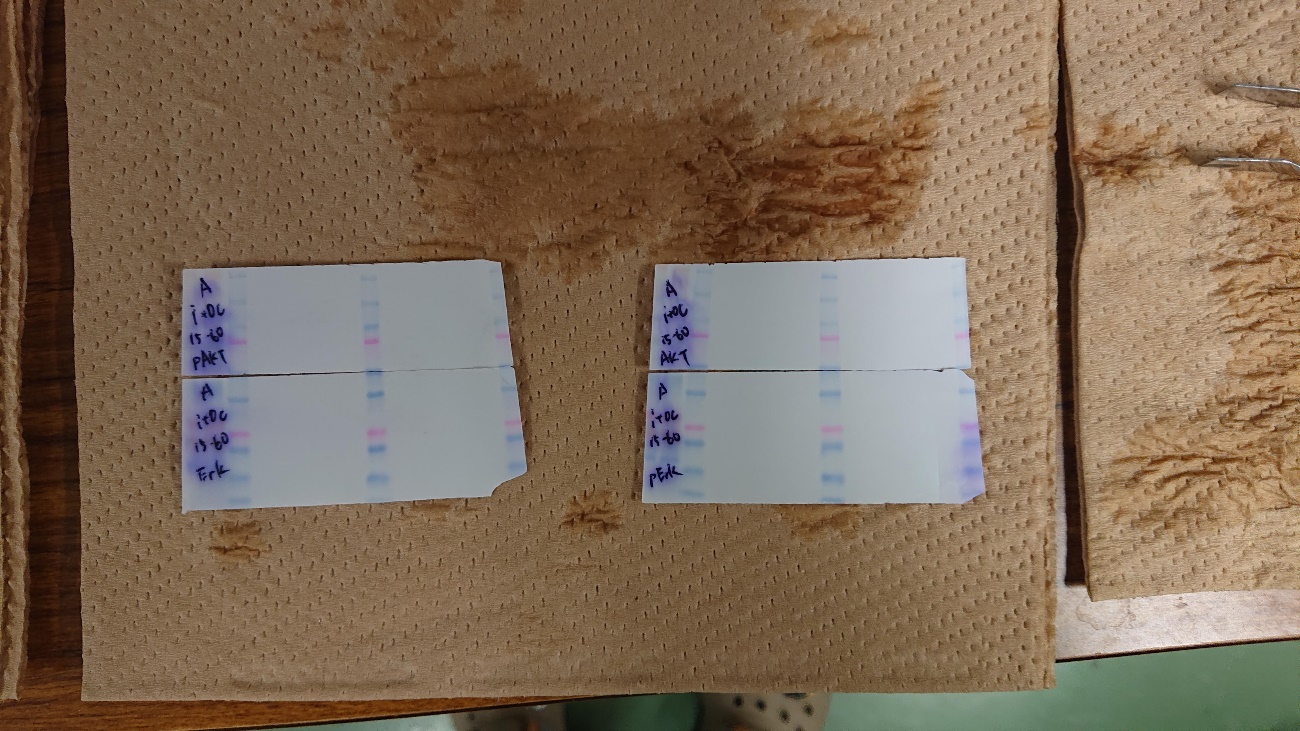


pAkt

tAkt

pErk

tErk

Multiple exposure images for pErk (Fig. 4e)


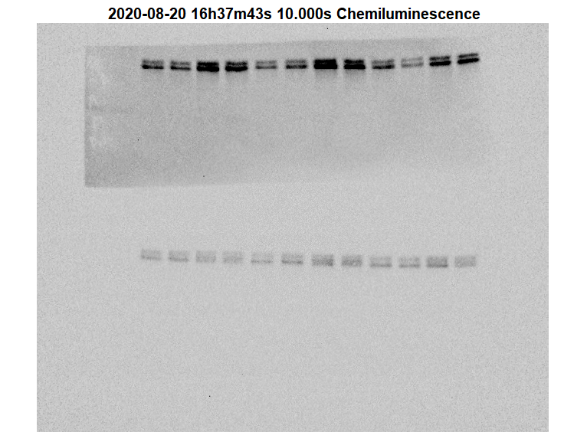

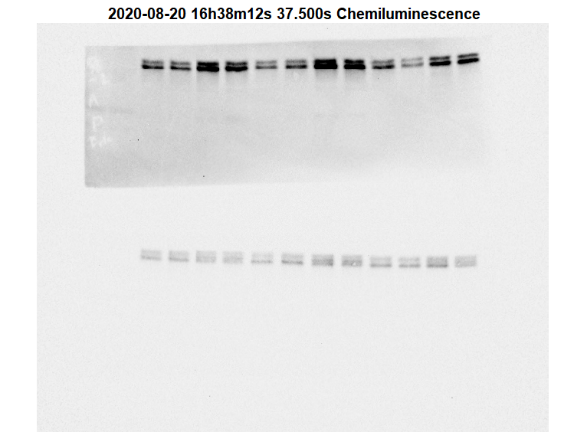


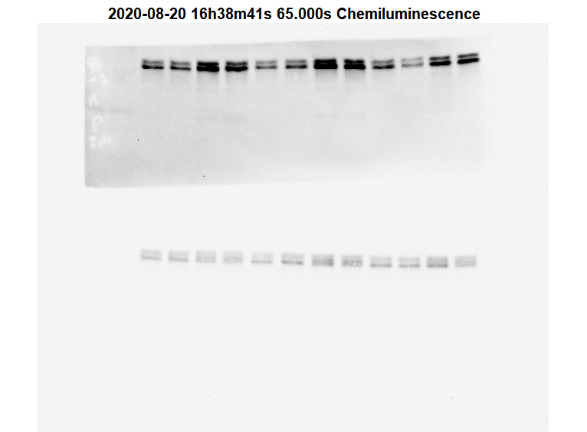

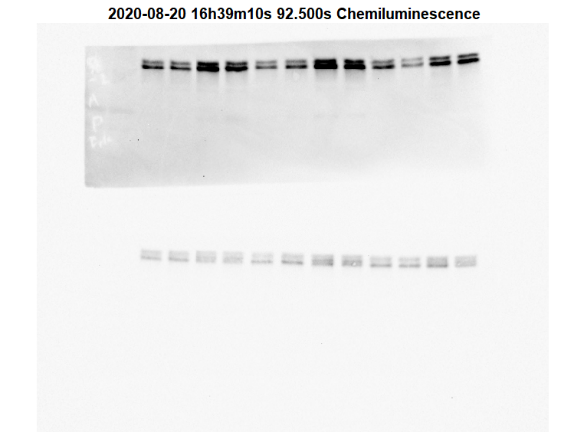


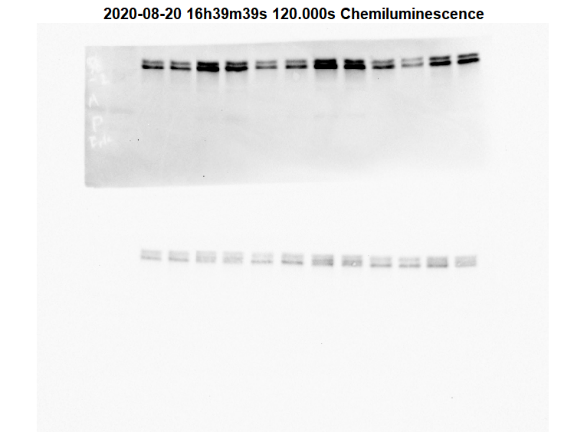


Multiple exposure images for tErk (Fig. 4e)


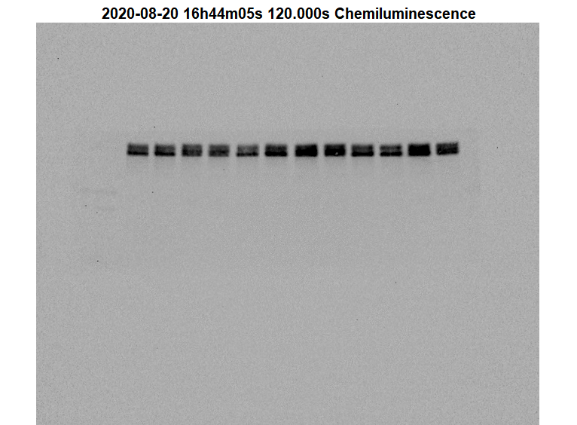

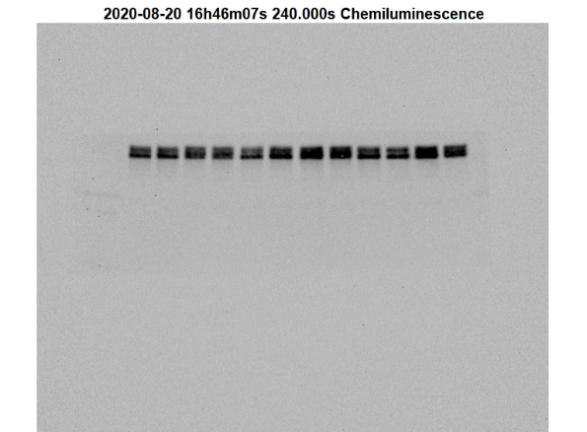


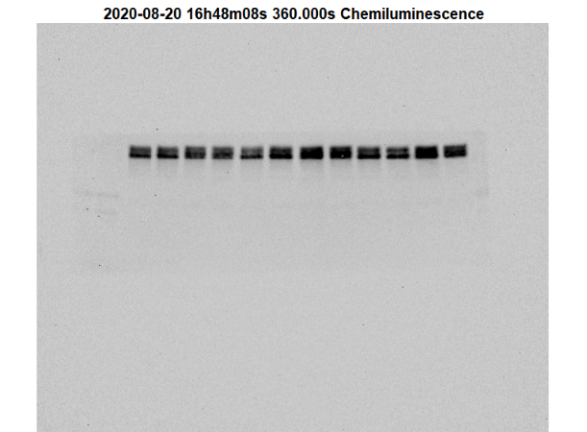

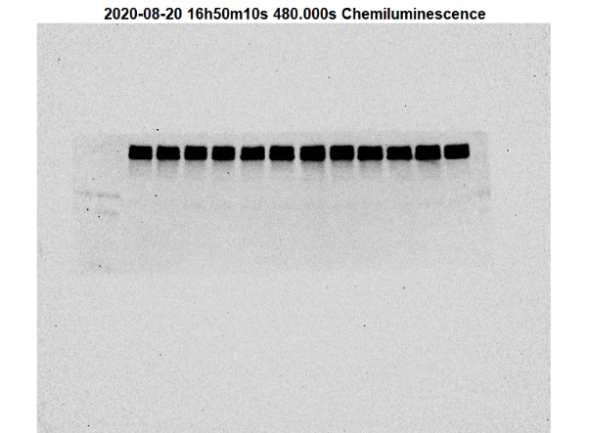


Multiple exposure images for pAkt/tAkt (Fig. 4e)


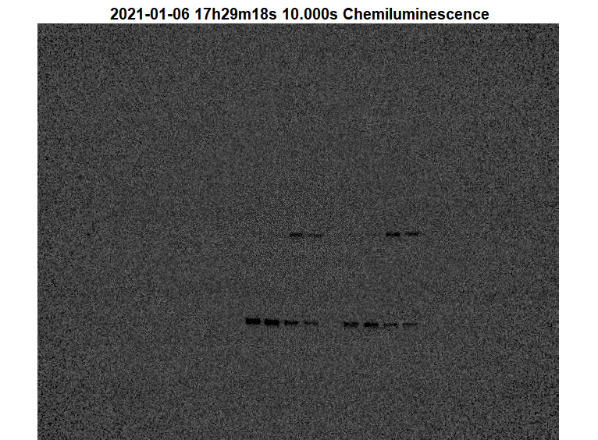

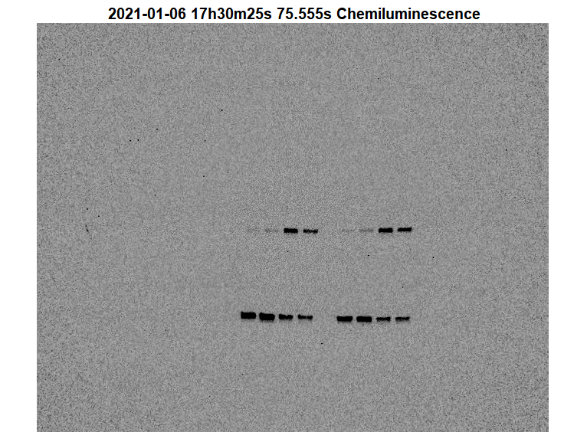


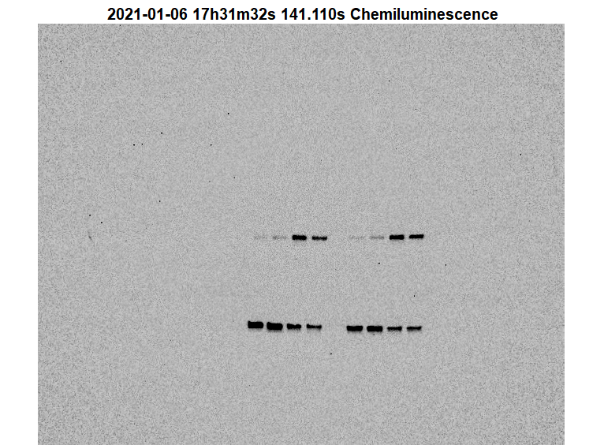

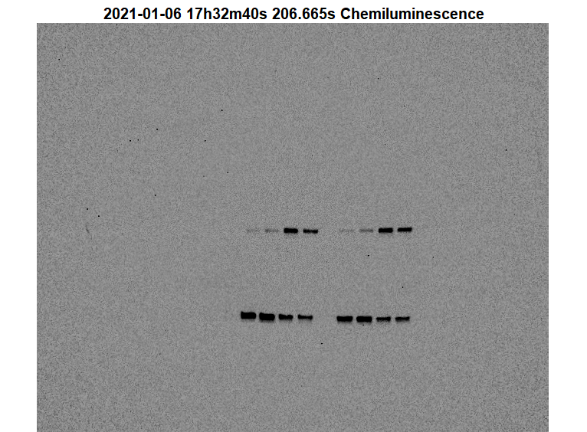


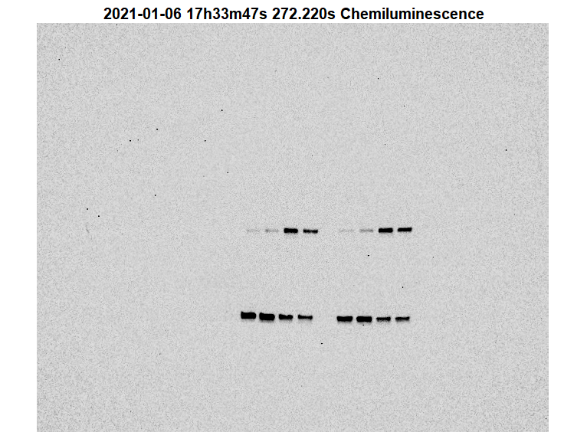

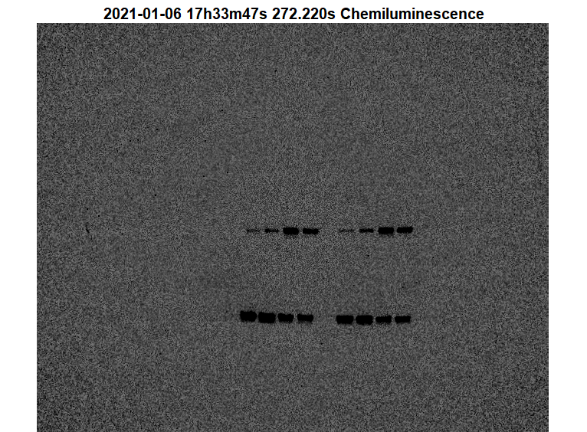


The blots placement during exposure for pAkt/tAkt.


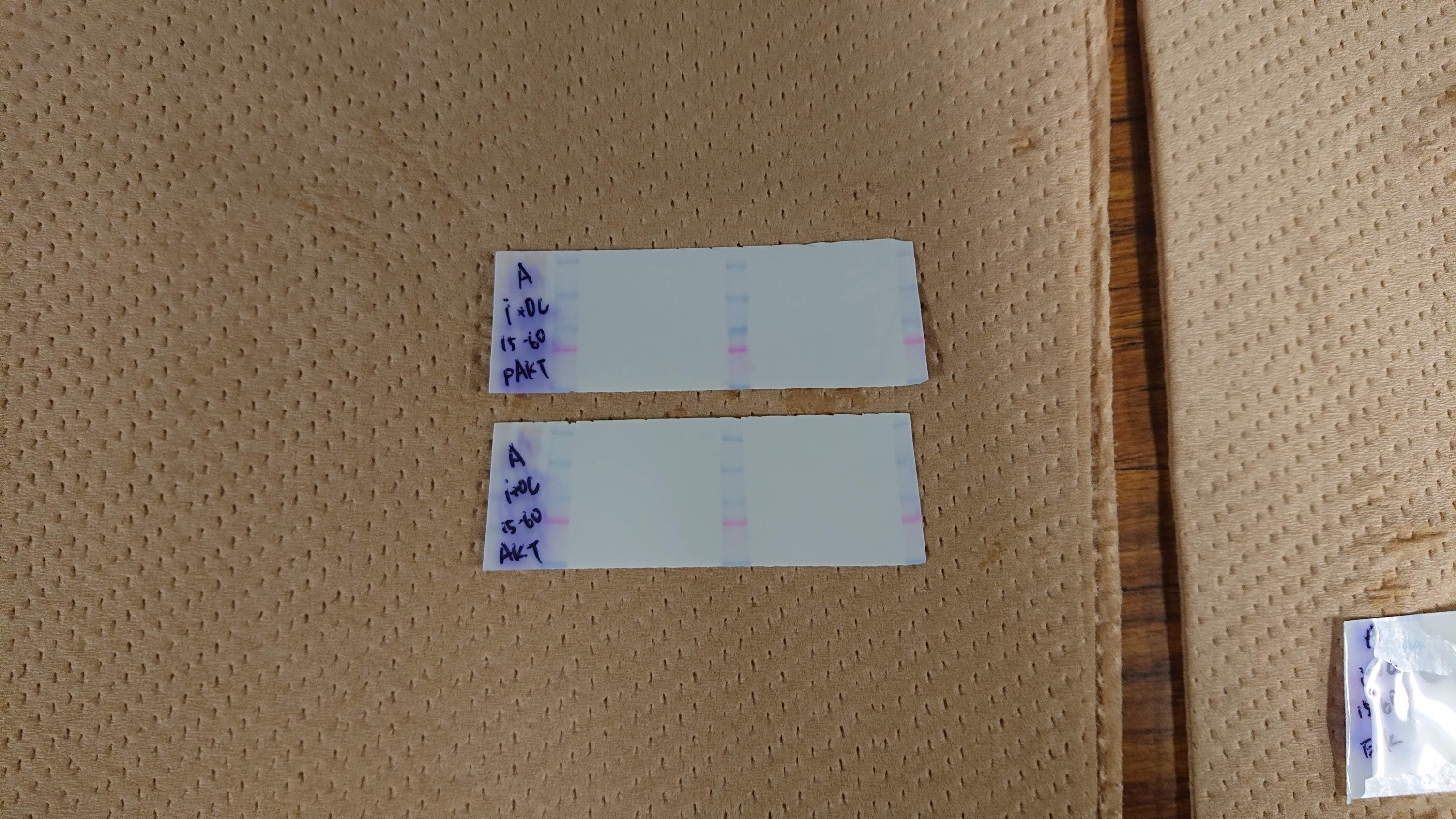


tAkt

pAkt

Fig. 4f


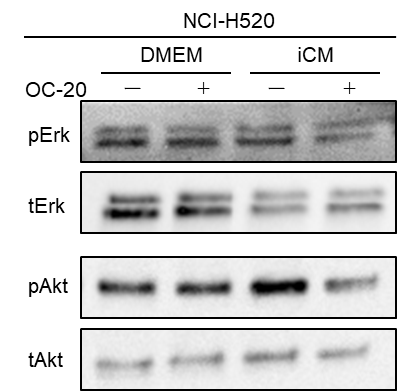


The membrane was cut prior to hybridization with antibodies. The top membranes were used for pAkt and tAkt, and the bottom membranes were used for pErk and tErk. Then, pErk and tErk, pAkt and tAkt were aligned up and down for visualization. The reason for this was to check the loading in one and also to make sure that each set does not shift.

pErk (Fig. 4f)


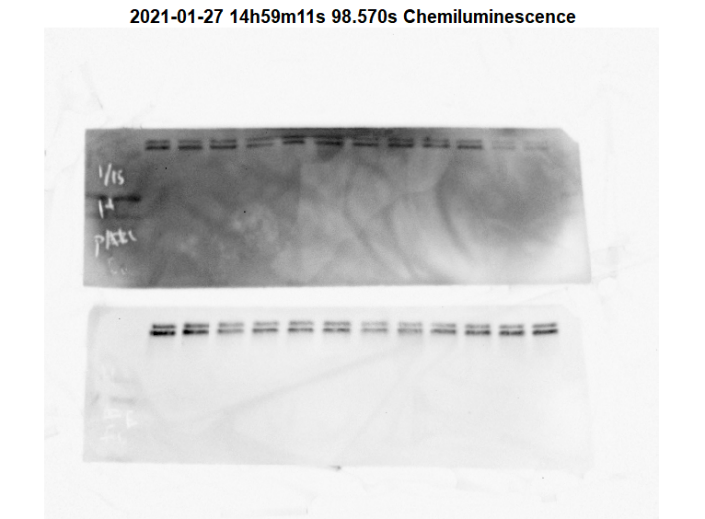


**pErk**

tErk (Fig. 4f)


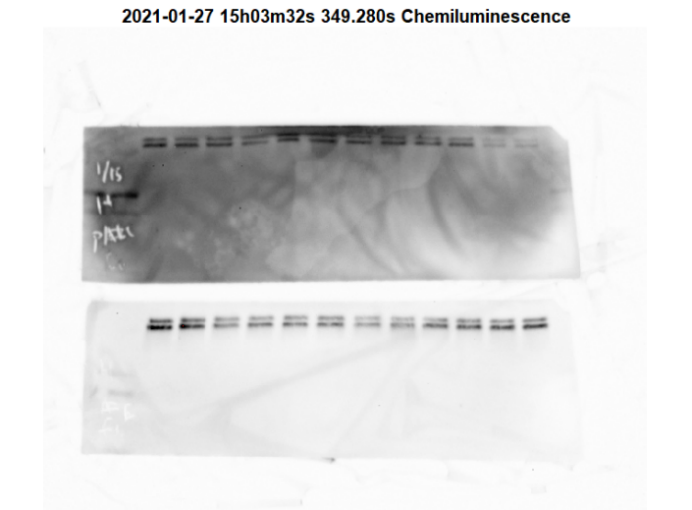


**tErk**

pAkt/tAkt (Fig. 4f)

The two images below are the same one with a different contrast, and the upper one is used for the figure.
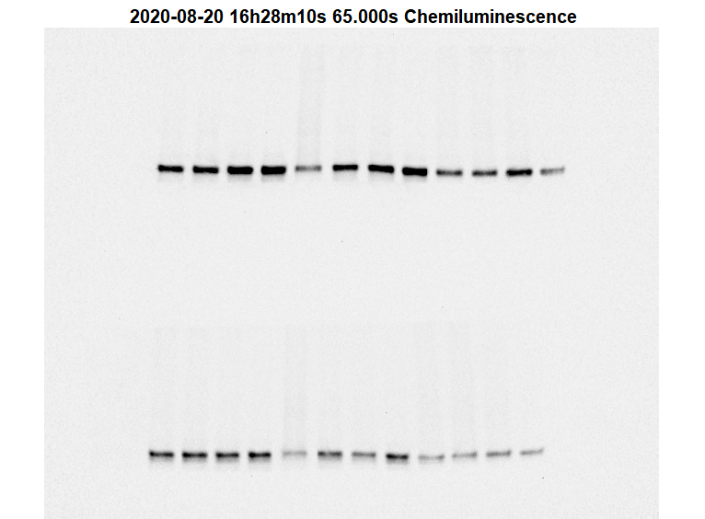

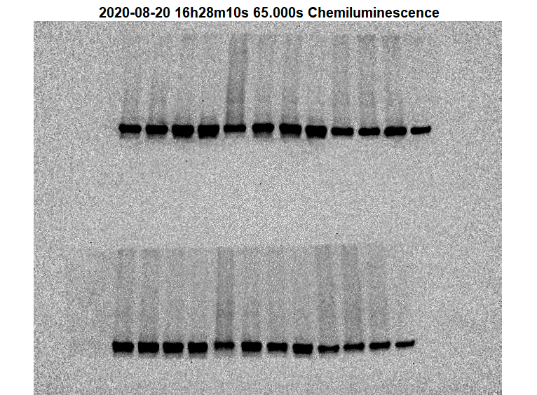


**pAkt**

**tAkt**

Supplemental fig. 1a


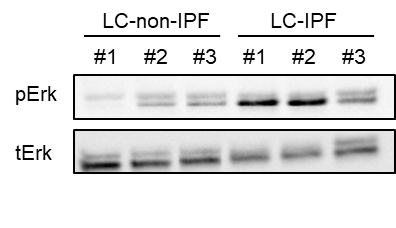


The membrane was cut prior to hybridization with antibodies.

The two images below are the same one with a different contrast, and the upper one was used for the figure for pErk.

pErk (Supplemental fig. 1a)


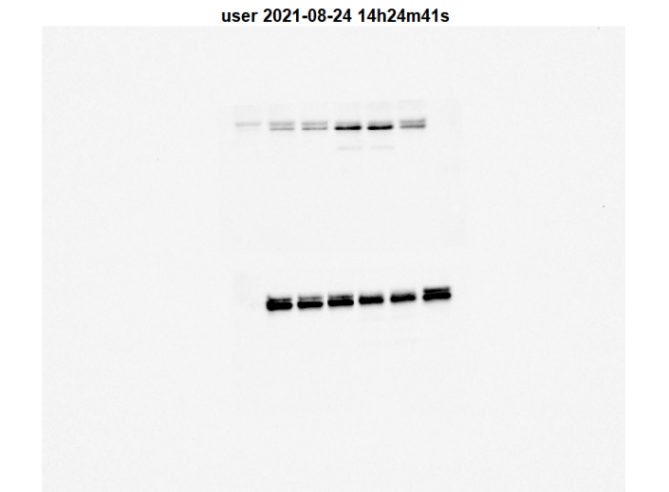

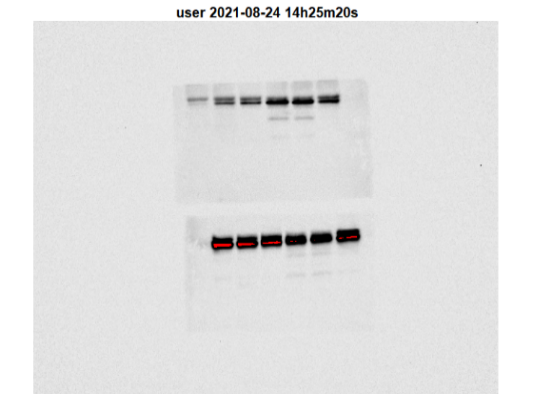


**pErk**

The two images below are the same one with a different contrast, and the upper one was used for the figure for tErk..

tErk (Supplemental fig. 1a)


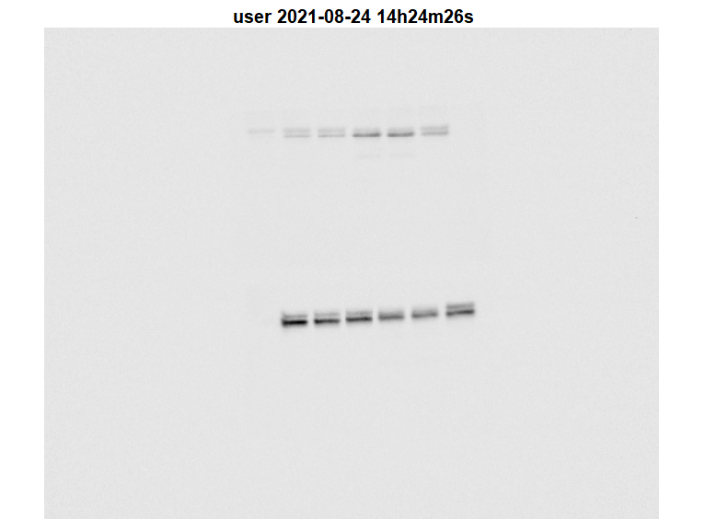

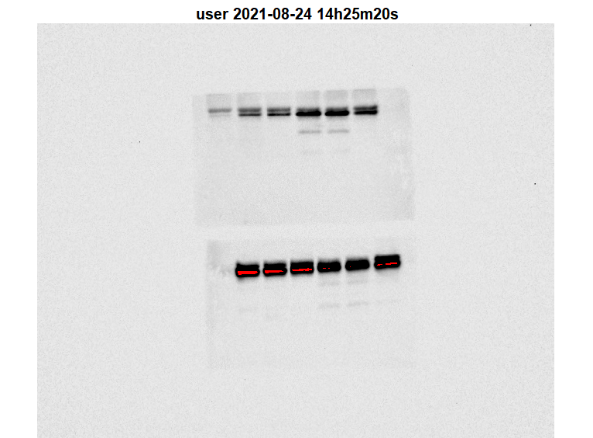


**tErk**

Supplemental fig. 3b


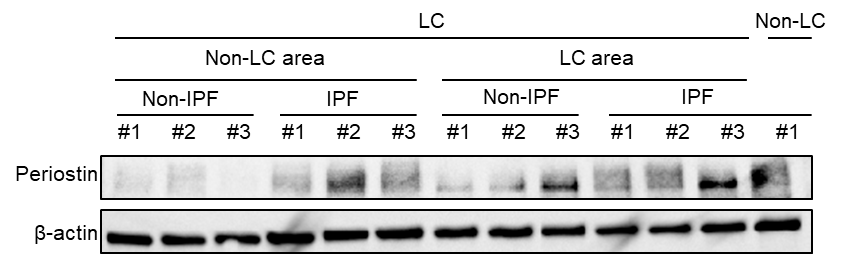


Periostin (Supplemental fig. 3b)


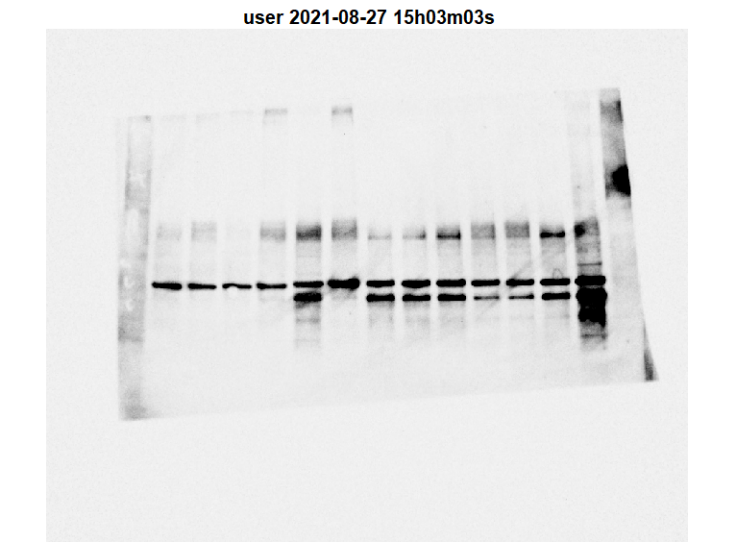


**Periostin**

β-actin (Supplemental fig. 3b)


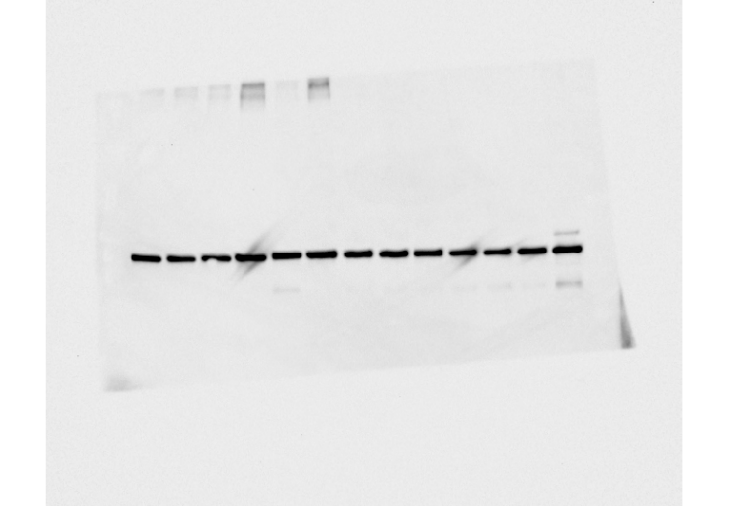


**Beta-actin**

Supplemental fig. 5a


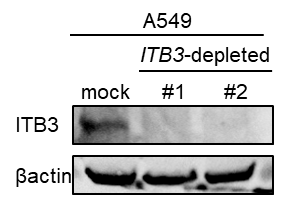


The membrane was cut prior to hybridization with antibodies.

Integrin beta3 (Supplemental fig. 5a)


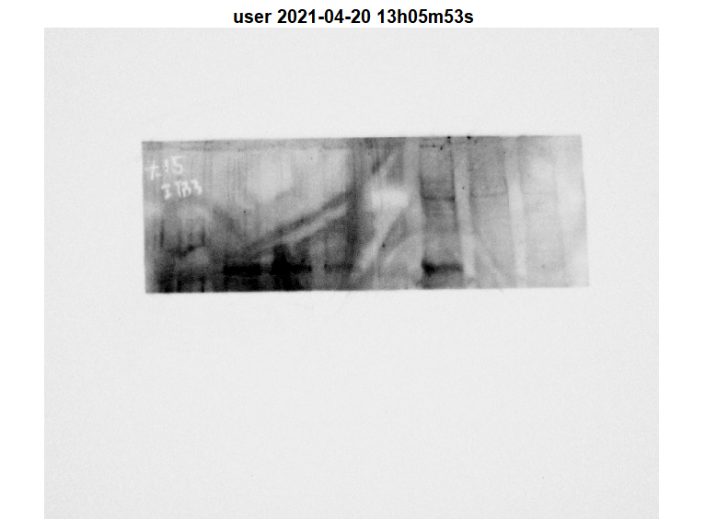


**Integrin beta3**

Beta-actin (Supplemental fig. 5a)


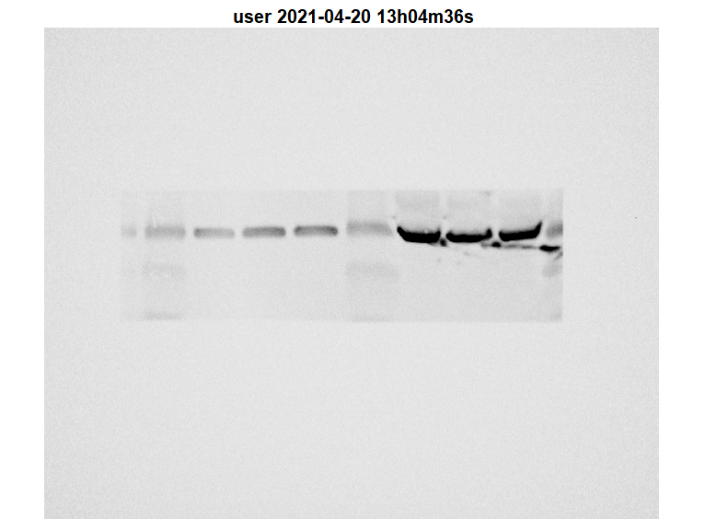


**Beta-actin**

Multiple exposure images for integrin beta3 (Supplemental fig. 5a)


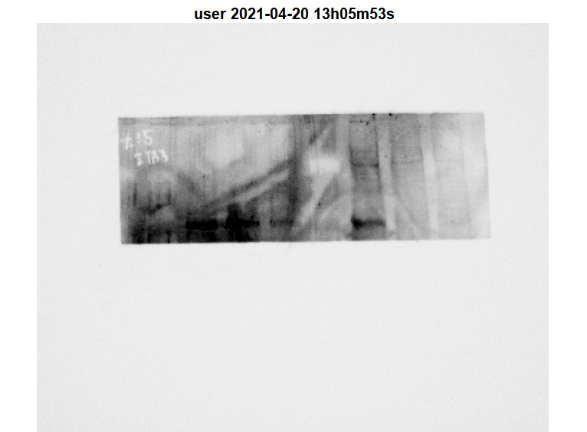

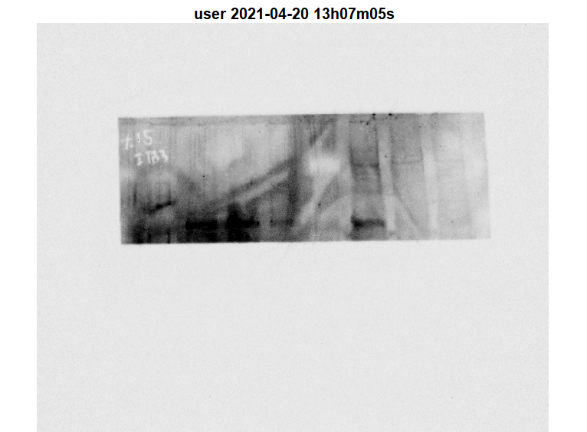


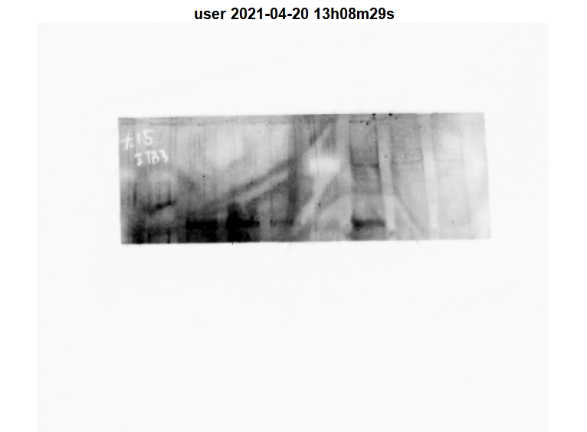

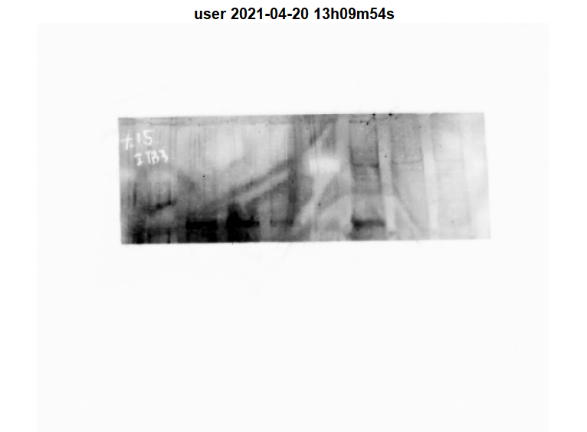


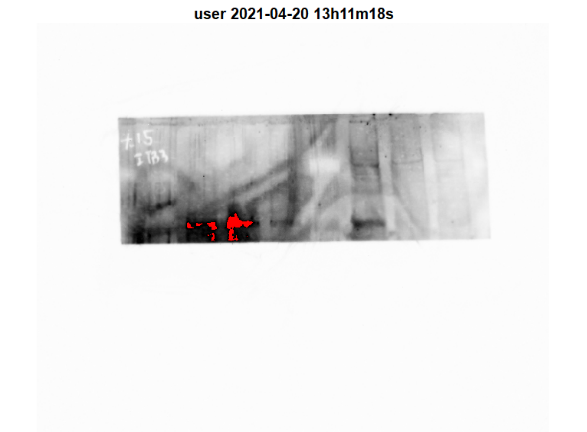

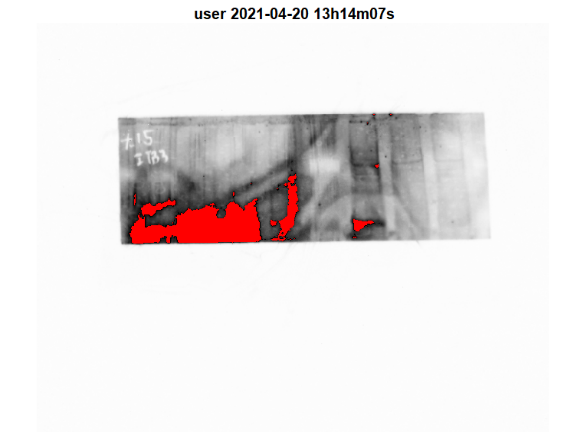

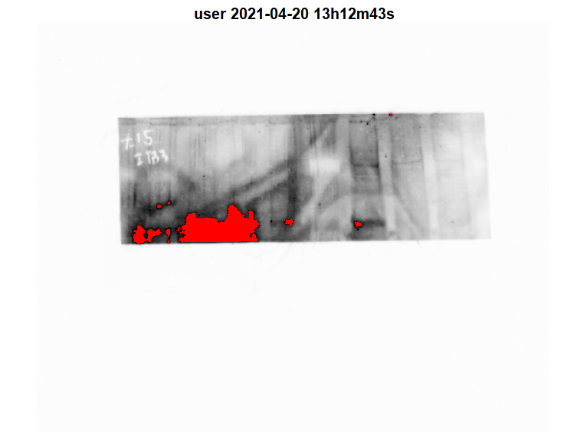

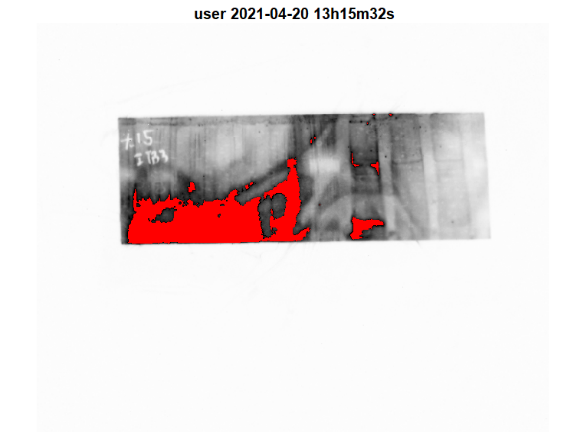


Multiple exposure images for beta-actin (Supplemental fig. 5a)


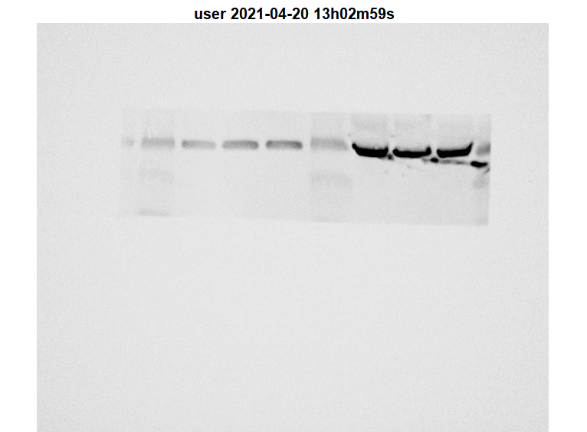

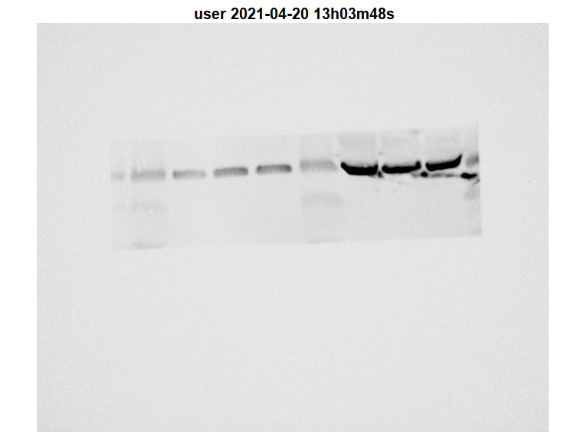


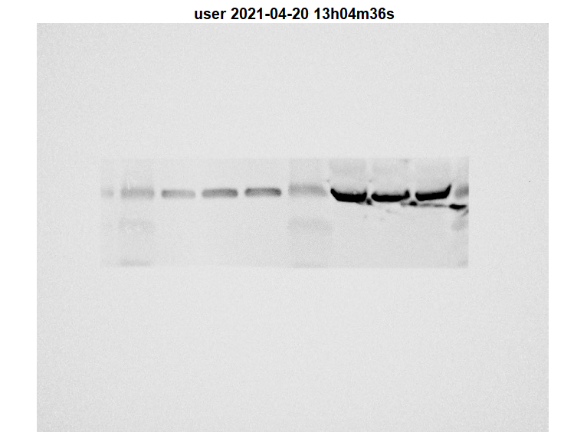


Supplemental fig. 5b


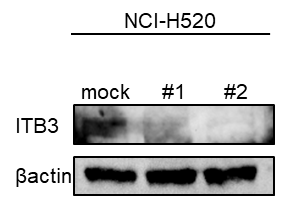


Integrin beta3 (Supplemental fig. 5b)


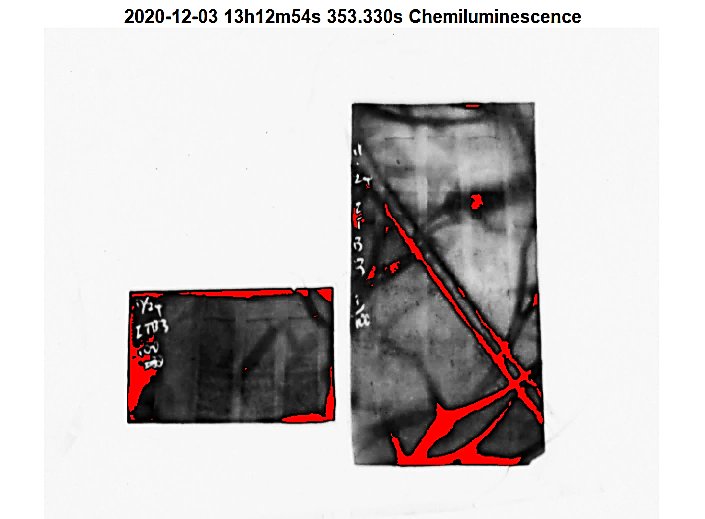


**Integrin beta3**

Beta-actin (Supplemental fig. 5b)

The membrane was cut prior to hybridization with antibodies.


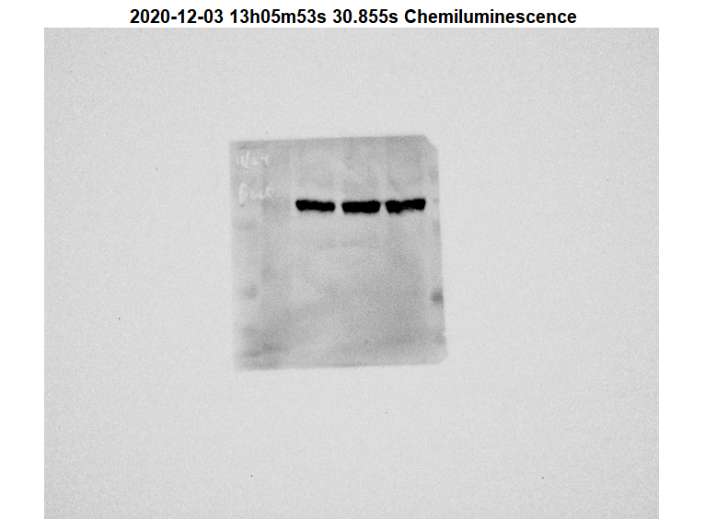


**Beta-actin**

bet
